# KAT6A Acetylation of SMAD3 Regulates Myeloid‐Derived Suppressor Cell Recruitment, Metastasis, and Immunotherapy in Triple‐Negative Breast Cancer

**DOI:** 10.1002/advs.202100014

**Published:** 2021-08-13

**Authors:** Bo Yu, Fei Luo, Bowen Sun, Wenxue Liu, Qiqi Shi, Shi‐Yuan Cheng, Ceshi Chen, Guoqiang Chen, Yanxin Li, Haizhong Feng

**Affiliations:** ^1^ State Key Laboratory of Oncogenes and Related Genes Renji‐Med X Clinical Stem Cell Research Center Ren Ji Hospital Shanghai Cancer Institute School of Medicine Shanghai Jiao Tong University Shanghai 200127 China; ^2^ Department of Neurology Lou and Jean Malnati Brain Tumor Institute The Robert H. Lurie Comprehensive Cancer Center Simpson Querrey Institute for Epigenetics Northwestern University Feinberg School of Medicine Chicago IL 60611 USA; ^3^ Key Laboratory of Animal Models and Human Disease Mechanisms of the Chinese Academy of Sciences and Yunnan Province Kunming Institute of Zoology Chinese Academy of Sciences Kunming 650223 China; ^4^ Key Laboratory of Pediatric Hematology and Oncology Ministry of Health Department of Hematology and Oncology Shanghai Children's Medical Center School of Medicine Shanghai Jiao Tong University Shanghai 200127 China

**Keywords:** immunotherapy, KAT6A, metastasis, myeloid‐derived suppressor cells, SMAD3, triple‐negative breast cancer

## Abstract

Aberrant SMAD3 activation has been implicated as a driving event in cancer metastasis, yet the underlying mechanisms are still elusive. Here, SMAD3 is identified as a nonhistone substrate of lysine acetyltransferase 6A (KAT6A). The acetylation of SMAD3 at K20 and K117 by KAT6A promotes SMAD3 association with oncogenic chromatin modifier tripartite motif‐containing 24 (TRIM24) and disrupts SMAD3 interaction with tumor suppressor TRIM33. This event in turn promotes KAT6A‐acetylated H3K23‐mediated recruitment of TRIM24–SMAD3 complex to chromatin and thereby increases SMAD3 activation and immune response‐related cytokine expression, leading to enhanced breast cancer stem‐like cell stemness, myeloid‐derived suppressor cell (MDSC) recruitment, and triple‐negative breast cancer (TNBC) metastasis. Inhibiting KAT6A in combination with anti‐PD‐L1 therapy in treating TNBC xenograft‐bearing animals markedly attenuates metastasis and provides a significant survival benefit. Thus, the work presents a KAT6A acetylation‐dependent regulatory mechanism governing SMAD3 oncogenic function and provides insight into how targeting an epigenetic factor with immunotherapies enhances the antimetastasis efficacy.

## Graphical abstract

TGF‐*β*1 potentiates KAT6A acetylation of SMAD3 at K20 and K117, which promotes SMAD3 association with oncogenic H3K23ac reader tripartite motif‐containing 24 (TRIM24) and thereby increases SMAD3 transcriptional activation. Activated SMAD3 upregulates immune‐related cytokines, leading to enhanced breast cancer stem‐like cell properties, myeloid‐derived suppressor cell (MDSC) recruitment, and triple‐negative breast cancer (TNBC) metastasis. Targeting KAT6A improves the anticancer metastasis efficacy of anti‐PD‐L1 therapy.

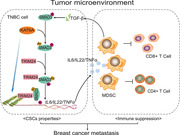

## 1 Introduction

Triple‐negative breast cancer (TNBC) is a particularly aggressive and metastatic breast cancer subtype.^[^ ^1^ ^]^ The high heterogeneity and the absence of well‐defined molecular targets renders TNBCs insensitive to endocrine‐targeted therapy and immunotherapy.^[^ ^1^, ^2^ ^]^ Despite having an initial response to presurgical (neoadjuvant) chemotherapy, TNBC patients have a higher rate of distant recurrence and a poorer prognosis than those with other breast cancer subtypes.^[^ ^3^ ^]^ Although several studies are ongoing, the underlying mechanisms for the aggressive phenotype and higher metastatic potential of TNBCs remain elusive.

Tumor microenvironment (TME) remodeling is a critical process for primary tumors to metastasize to distant organs.^[^ ^4^ ^]^ Myeloid‐derived suppressor cells (MDSCs) are a major component of the TME, and their increased number is associated with a poor prognosis in various cancers.^[^ ^5^, ^6^ ^]^ MDSCs are produced in the bone marrow of tumor‐bearing hosts and recruited to the primary tumor and metastatic niches to support metastasis in response to tumor‐derived chemokines and hypoxia‐induced factors.^[^ ^7^ ^]^ While the functions of MDSCs in breast cancer have been evaluated in mouse models and patients, the mechanistic basis for MDSCs recruitment is still unknown.

Lysine acetyltransferase 6A (KAT6A, also known MYST3 or MOZ) belongs to the MYST‐family of histone acetyltransferases (HATs) and is a chromatin modifier with acetyltransferase activity to acetylate histone and nonhistone proteins.^[^ ^8^ ^]^ KAT6A and KAT6B (also known as MORF) forms a tetrameric complex with Inhibitor of growth 5 (ING5), Esa1‐associated factor 6 ortholog (EAF6), and the bromodomain‐PHD finger protein BRPF1 to regulate gene transcription by acetylating histones near target gene promoters or enhancers or recruiting acetylated histone reader proteins, such as tripartite motif‐containing 24 (TRIM24).^[^ ^9^, ^10^, ^11^, ^12^ ^]^ While KAT6B is considered a tumor suppressor,^[^ ^13^ ^]^ KAT6A exhibits a predominantly oncogenic function in a number of cancers, including leukemia, glioma, endometrial serous carcinoma, and breast cancer.^[^ ^10^, ^12^, ^14^, ^15^ ^]^ In acute myeloid leukemia, KAT6A undergoes changes including gene mutation and fusion with proteins resulting from chromosomal rearrangement.^[^ ^16^, ^17^, ^18^, ^19^ ^]^ Earlier studies showed that KAT6A is amplified and/or overexpressed in Luminal A, Luminal B, HER2^+^, and TNBC/basal‐like subtype breast cancers, and its overexpression correlates with worse clinical outcome in ER^+^/HER2^−^ breast cancers.^[^ ^20^ ^]^ However, our current understanding of KAT6A function in breast cancer metastasis and TME is still limited.

SMAD3 is a member of the SMAD family of proteins that acts as a mediator of TGF‐*β* superfamily‐modulated signaling that regulates cell proliferation, apoptosis, immune surveillance, and cancer metastasis.^[^ ^21^ ^]^ Phosphorylated SMAD3 by the TGF‐*β* receptors and/or SMAD2 forms an oligomeric complex with SMAD4.^[^ ^22^ ^]^ This complex is transported into the nucleus, where SMADs bind to promoters of target genes such as interleukin 6 ( (*IL‐6)* and *CXCL2*, which recruit MDSCs to promote metastasis.^[^ ^7^, ^23^, ^24^ ^]^ SMAD3 activity is tightly regulated by ubiquitination, SUMOylation, acetylation, and phosphorylation in cancers.^[^ ^21^ ^]^ However, the role of SMAD3 in cancer metastasis has yet to be demonstrated. In this study, using mass spectrometry (MS) analysis, we identified SMAD3 as a new nonhistone substrate of KAT6A. Given that both KAT6A and TGF‐*β*/SMAD3 are activated in many cancers, including breast cancer, we investigated potential crosstalk between these two intensively studied oncogenic pathways and determined the biological consequence of the KAT6A–SMAD3 signaling in TNBC metastasis.

## 2 Results

### 2.1 The Amplification and/or Overexpression of *KAT6A* Are Associated with TNBC Metastasis

**Figure 1:**
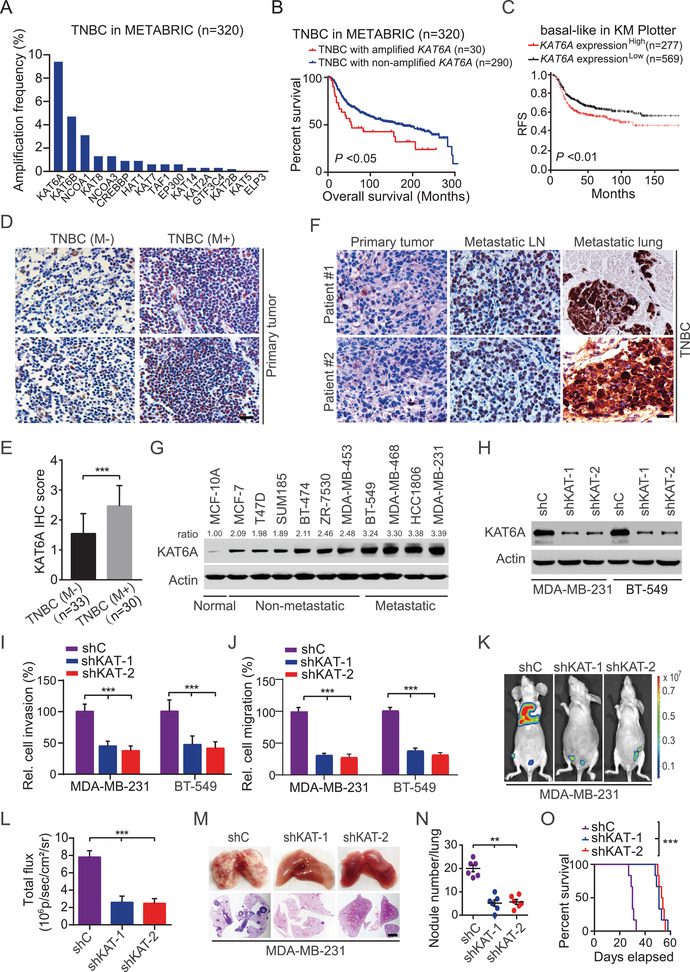
The amplification and/or overexpression of *KAT6A* are associated with triple‐negative breast cancer (TNBC) metastasis. A) DNA copy‐number variation (CNV) analysis of 16 lysine acetyltransferase (KAT) genes in TNBC specimens from the Molecular Taxonomy of Breast Cancer International Consortium (METABRIC) dataset (*n* = 320). B) Kaplan–Meier survival analysis of TNBC patients with *KAT6A* amplification in panel (A). Median survival (in months): amplified, 55.6; nonamplified, 175.1. Black bars, censored data. C) High KAT6A expression correlates with reduced relapse‐free survival (RFS) in TNBC/basal‐like specimens in the Kaplan–Meier plotter dataset. Cutoff value used in analysis, 61. D) Representative IHC staining of KAT6A in primary tumors of TNBC with (M+) or without (M−) distant organ metastasis. Two different TNBC specimens with (M+) and two different TNBC specimens without (M−). Scale bars: 50 µm. E) Quantification of KAT6A expression in panel (D). F) Representative IHC staining of KAT6A protein in primary tumors and paired lymph node (LN) as well as lung metastases. Scale bars: 50 µm. G) western blotting (WB) of KAT6A protein expression in nontumorigenic epithelial cell line MCF‐10A, metastatic, and nonmetastatic breast cancer cell lines. H) WB of *KAT6A* knockdown (KD) using two different *KAT6A* shRNAs (shKAT‐1 and shKAT‐2) or a control shRNA (shC). I) Effects of *KAT6A* depletion on MDA‐MB‐231 and BT‐549 cells invasion. J) The effect of *KAT6A* depletion on cell migration in MDA‐MB‐231 and BT‐549 cells. K) Representative bioluminescence images of *KAT6A* KD‐decreased tumor metastasis (*n* = 6). Mice were imaged at 4–5 weeks after tail vein injection. L) Quantification of the bioluminescence activity in panel (K). M) Representative bright‐field (upper panel) and hematoxylin and eosin (H&E) staining (lower panel) imaging of the lungs in panel (K). Scale bars: 100 µm. N) The number of macroscopic lesions on the lung surfaces in panel (K) were quantified at necropsy. O) Kaplan–Meier survival analysis of animals with indicated MDA‐MB‐231 tumors (*n* = 6). In panels (D) to (O), data are representative of three independent experiments with similar results. Error bars, SEM. *P < 0.05, **P < 0.01, and ***P < 0.001, by paired two‐tailed *t*‐test or log‐rank test.

To identify the role of KAT6A in breast cancer metastasis, we first examined the DNA copy numbers of all KATs in clinical TNBC specimens from the Molecular Taxonomy of Breast Cancer International Consortium (METABRIC) dataset (https://www.cbioportal.org/).^[^ ^25^ ^]^ KAT genes show various degrees of copy‐number amplification in TNBC, and *KAT6A* is mostly amplified (30/320, 9.4%) (**Figure** **1**A). Kaplan–Meier analysis showed that TNBC patients with *KAT6A* amplification (median survival, 55.6 months) had a worse overall survival compared with those with non‐*KAT6A* amplification (median survival, 175.1 months) (Figure 1B). In addition, high levels of KAT6A significantly correlate with reduced relapse‐free survival (RFS) in the basal clinical breast cancer samples in the Kaplan–Meier Plotter dataset (https://kmplot.com/analysis) (Figure 1C).

Next, we examined the protein levels of KAT6A in TNBC tumors with or without distant metastasis by immunohistochemical (IHC) staining. As shown in Figure 1D,E, TNBC tumors with distant metastasis showed a remarkably higher KAT6A expression than those without tumor metastasis. Compared to the paired primary TNBC tumors, KAT6A was dramatically upregulated in lymph node and lung metastasis in all of the five patients (Figure 1F; Figure S1A, Supporting Information).

We further assessed KAT6A expression in a nontumorigenic epithelial cell line MCF‐10A and various breast cancer cell lines and revealed that compared with MCF‐10A cells, KAT6A was highly expressed in all tested breast cancer cells (Figure 1G). In addition, the level of KAT6A protein was higher in TNBC cells with highly metastatic ability than that in those nonmetastatic cells (Figure 1G). Knockdown (KD) of *KAT6A* with two different shRNAs significantly decreased cell proliferation (Figure 1H; Figure S1B, Supporting Information) in MDA‐MB‐231 and BT‐549 cells and orthotopic xenograft tumor growth (Figure S1C,D, Supporting Information). Moreover, *KAT6A* KD decreased cell invasion and migration in MDA‐MB‐231 and BT‐549 cells (Figure 1I,J) and tumor metastasis to the lungs (Figure 1K,L). *KAT6A* KD markedly inhibited metastatic lung nodule formation (Figure 1M,N) and prolonged animal survival (Figure 1O). These data demonstrate that KAT6A is critical for TNBC metastasis and patients with amplified *KAT6A* have a poor prognosis.

### 2.2 KAT6A Acetylates SMAD3 at Lys20 and Lys117

**Figure 2:**
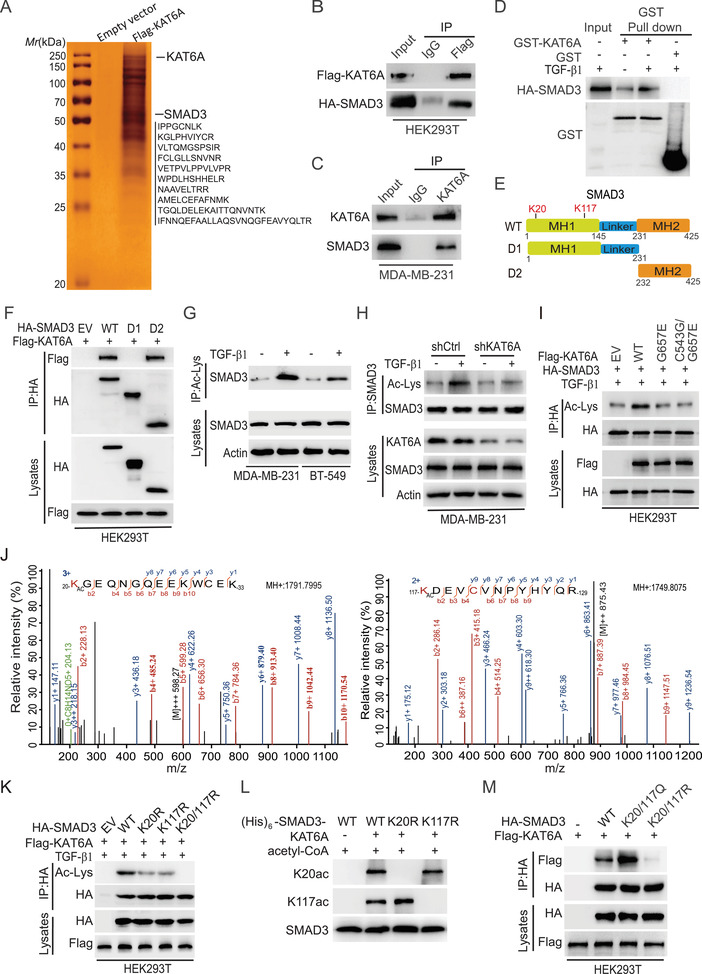
KAT6A acetylates SMAD3 at K20 and K117. A) Immunoprecipitation (IP) and mass spectrometry (MS) analyses of KAT6A‐associated proteins in MDA‐MB‐231 cells. B) Co‐immunoprecipitation (Co‐IP) of exogenous KAT6A with SMAD3 in HEK293T cells. C) Co‐IP of endogenous KAT6A with SMAD3 in MDA‐MB‐231 cells. D) In vitro GST pull‐down analysis. Purified GST‐KAT6A or GST proteins were incubated with cell extracts from MDA‐MB‐231 cells stimulated with or without TGF‐*β*1 (5 ng mL^−1^, 2 h). E) Schematics of SMAD3 wild type (WT), D1 (MH1 and Linker domain) mutant, and D2 (MH2 domain) mutant. F) IP and WB of KAT6A interaction with SMAD3 WT or mutants. EV, an empty vector control. G) IP and WB for analyzing the acetylated SMAD3 in MDA‐MB‐231 and BT‐549 cells treated with or without TGF‐*β*1 (5 ng mL^−1^, 2 h). A pan acetylation antibody (9441S, CST) was used. H) *KAT6A* knockdown (KD) decreases the acetylation (Ac‐Lys) of SMAD3 in MDA‐MB‐231 cells. I) Effect of acetyltransferase activity‐deficient mutants G657E and C543G/G657E of KAT6A on SMAD3 acetylation. J) MS analysis of acetylation sites of SMAD3 by KAT6A. In vitro acetylated SMAD3 by KAT6A as in panel (D) was purified and then subjected to MS analysis. K) Effect of K20R, K117R, or K20/117R mutation of SMAD3 on KAT6A‐mediated SMAD3 acetylation. L) In vitro KAT analysis using recombinant active KAT6A and (His)6‐SMAD3 WT, K20R, or K117R mutant protein. Acetylation of K20 or K117 was determined by using anti‐K20ac or anti‐K117ac antibodies, respectively. M) IP and WB of KAT6A association with WT SMAD3, the acetylation‐mimetic K20/117Q, or acetylation‐deficient K20/117R mutant. Data are representative of three independent experiments with similar results.

To explore the putative targets regulated by KAT6A in breast cancer metastasis, we purified the KAT6A complex from MDA‐MB‐231 cells transduced with Flag‐tagged KAT6A and performed MS analysis. As shown in **Figure** **2**A and Table S1 in the Supporting Information, among the top ten of putative KAT6A‐binding proteins identified in our analysis, SMAD3 was highly enriched in the KAT6A precipitate and ten peptides of SMAD3 were identified in the protein complex (peptide score >45, peptide expect <0.01). Since SMAD3 is critical for breast cancer metastasis,^[^ ^26^ ^]^ we chose SMAD3 as a major candidate for KAT6A regulation of breast cancer metastasis.

We validated KAT6A interaction with SMAD3 by immunoprecipitation (IP) and western blotting (WB) assays in HEK293T (Figure 2B). This was further confirmed with endogenous KAT6A and SMAD3 protein in MDA‐MB‐231 cells (Figure 2C). We then carried out glutathione S‐transferase (GST) pull‐down analysis and found that purified recombinant KAT6A directly interacted with SMAD3, and TGF‐*β*1 stimulation significantly enhanced KAT6A–SMAD3 association (Figure 2D). IP‐WB analyses of HEK293T cells that KAT6A was coexpressed with wild type (WT) SMAD3 or the deletion mutants further revealed that the C‐terminal MH2 domain (amino acids 232–425) of SMAD3 is required for its association with KAT6A (Figure 2E,F).

Next, we determined whether SMAD3 is a substrate of KAT6A by assessing the effect of *KAT6A* KD on SMAD3 acetylation. First, we determined and found that TGF‐*β*1‐stimulated SMAD3 acetylation (Figure 2G), which was consistent with previous reports^[^ ^27^, ^28^ ^]^ and then KD of *KAT6A* significantly reduced TGF‐*β*1‐stimulated SMAD3 acetylation (Figure 2H). In addition, compared to WT KAT6A, the KAT6A acetyltransferase activity‐deficient mutants, G657E or C543G/G657E,^[^ ^17^, ^20^ ^]^ markedly attenuated SMAD3 acetylation (Figure 2I).

To identify the putative acetylation of lysine (K) residues of SMAD3 by KAT6A, we purified KAT6A‐associated SMAD3 and performed MS analysis. As shown in Figure 2J, lysine 20 (K20) and lysine 117 (K117) on the MH1 domain of SMAD3 were found acetylated, and these two residues were highly conserved across species (Figure S2A, Supporting Information). Mutation of either lysine to arginine (K to R) decreased KAT6A‐induced SMAD3 acetylation with TGF‐*β*1 stimulation, and double KR mutations abrogated the acetylation of SMAD3 (Figure 2K). To further confirm the KAT6A acetylation of SMAD3, we generated two rabbit polyclonal antibodies that specifically recognized SMAD3‐K20ac or SMAD3‐K117ac through a commercial vendor and validated their specificity in a clinical breast cancer specimen and MDA‐MB‐231 cells (Figure S2B–D, Supporting Information). In vitro KAT assay using purified recombinant active KAT6A and recombinant WT SMAD3 or the K20R or K117R mutant confirmed the acetylation of K20 and K117 by KAT6A (Figure 2L). Moreover, compared to WT SMAD3, the acetylation‐mimic K20/117Q mutant enhanced SMAD3 association with KAT6A in HEK293T cells, whereas the nonacetylatable K20/117R mutant inhibited their interaction (Figure 2M). Additionally, we assessed the effects of depletion of *KAT6B*, *p300*, *KAT5*, *KAT2B*, or *KAT2A* using sgRNAs on acetylation of K20 and K117 of SMAD3 and identified that KAT6A but not other KATs specifically acetylated SMAD3 at these two lysine residues (Figure S2E, Supporting Information). Coexpression of KAT6A with WT SMAD3 or the D1 deletion mutant further showed that the C‐terminal MH2 domain (the D2 mutant) of SMAD3 is required for KAT6A acetylation of K20 and K117 (Figure S2F, Supporting Information). In addition, *KAT6A* KD markedly reduced the levels of SMAD3 K20ac and K117ac in MDA‐MB‐231 and BT‐549 cells (Figure S2G, Supporting Information), metastatic lung tissues, and primary xenograft tumors (Figure S2H,I, Supporting Information). Moreover, forced overexpression of KAT6A‐WT enhanced the levels of SMAD3 K20ac and K117ac, and further promoted cell proliferation, migration, and invasion in MDA‐MB‐231/sgSMAD3 and BT‐549/sgSMAD3 cells with re‐expressing SMAD3 WT but not the K20/117R mutant (Figure S2J–L, Supporting Information). Therefore, these data indicated that KAT6A could directly acetylate SMAD3 at the K20 and K117 residues.

Since SMAD3 activation depends on its phosphorylation stimulated by TGF‐*β*1^[^ ^29^, ^30^ ^]^ and KAT6A localizes in the nucleus (Figure 1D,F), we investigated whether TGF‐*β*1‐induced phosphorylation of SMAD3 affects SMAD3 acetylation. As shown in Figure S3A in the Supporting Information, TGF‐*β*1 induced SMAD3 phosphorylation (p‐SMAD3) at the indicated time points in MDA‐MB‐231 cells. K20ac and K117ac of SMAD3 were also enhanced by TGF‐*β*1 stimulation with a pattern similar to p‐SMAD3 (Figure S3A, Supporting Information). Treatment of SB431542, a potent and selective inhibitor of the TGF‐*β* type I receptor, markedly inhibited TGF‐*β*1‐stimulated p‐SMAD3 as well as K20ac and K117ac (Figure S3B, Supporting Information). Compared with WT SMAD3, ectopic expression of the K20/117R mutant impaired SMAD3 acetylation but not p‐SMAD3 in MDA‐MB‐231 cells (Figure S3C, Supporting Information). Moreover, deletion of *KAT6A* in immortalized *Kat6a^fl/fl^* mouse embryonic fibroblasts (MEFs) abolished the effect of TGF‐*β*1 on SMAD3 K20ac and K117ac but not p‐SMAD3 (Figure S3D, Supporting Information), and *KAT6A* KD in MDA‐MB‐231 and BT‐549 cells inhibited the TGF‐*β* induced expression of PAI‐1, a prototypic TGF‐*β*/SMAD3 signaling target gene (Figure S3E, Supporting Information). In addition, in *SMAD3* knock‐out (KO) MDA‐MB‐231 cells, ectopic expression of SMAD3 WT or the K20/117Q mutant can rescue *SMAD3* KO‐inhibited p‐SMAD3 stimulated by TGF‐*β*1, whereas only the K20/117Q mutant markedly induced PAI‐1 expression without TGF‐*β*1 stimulation compared to the SMAD3 WT (Figure S3F, Supporting Information), suggesting that without TGF‐*β*1 stimulation, the K20/117Q mutant has more potential to activate SMAD3 signaling compared to the WT. To further demonstrate this, we performed subcellular localization analysis and found that compared with SMAD3 WT and 2KR mutant, the 2KQ mutant mainly localized in the nucleus without TGF‐*β*1 stimulation (Figure S3G, Supporting Information). After TGF‐*β*1 treatment, the nuclear localization of SMAD3 WT, 2KR, and 2KQ mutants all significantly increased. SMAD3 2KQ mutation significantly delayed nuclear export compared to the WT (Figure S3H, Supporting Information) after T*β*RI inhibitor SB431542 treatment whereas the 2KR mutation increased nuclear export compared to the WT (Figure S3I, Supporting Information). In addition, in vitro KAT assay using purified recombinant active KAT6A and recombinant SMAD3 WT, the phosphorylation‐mimetic 2SE (SSVS→SEVE, Ser423/425Ala) or phosphorylation‐deficient 2SA (SSVS→SAVA, Ser423/425Glu) mutant^[^ ^31^, ^32^ ^]^ confirmed that the phosphorylation status of SMAD3 had no directly effect on the acetylation of K20 and K117 of SMAD3 by KAT6A (Figure S3J, Supporting Information). Taken together, these data suggest that SMAD3 is a substrate for KAT6A‐mediated acetylation and TGF‐*β*1‐induced phosphorylation of SMAD3 promotes SMAD3 nuclear translocation, which facilities SMAD3 acetylation by KAT6A.

### 2.3 The K20/K117 Acetylation of SMAD3 Upregulates Immune Response‐Related Cytokines

**Figure 3:**
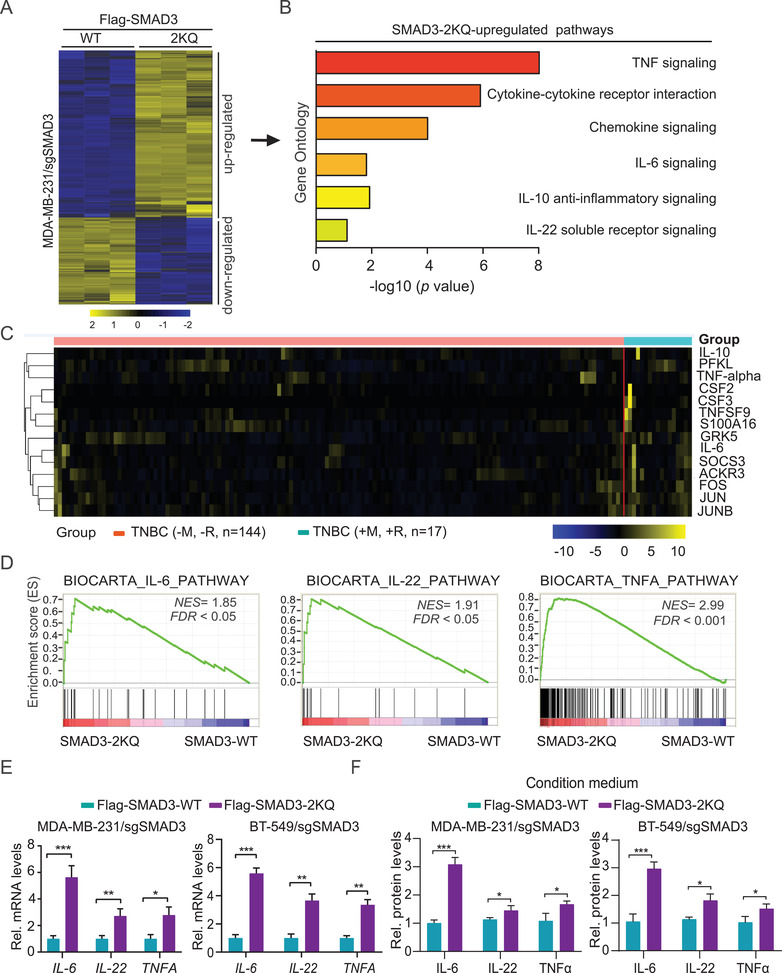
K20/K117 acetylation of SMAD3 upregulates immune response‐related cytokines. A) Heatmap of mRNA‐Seq analysis of differentially expressed genes regulated by SMAD3 K20/117Q (SMAD3‐2KQ) (twofold change and FDR < 0.05). SMAD3 sgRNA‐resistant Flag‐SMAD3 WT and K20/117Q (2KQ) mutant were infected into sgSMAD3/MDA‐MB‐231 cells without TGF‐*β*1 treatment. B) Gene ontology (GO) analysis indicates that the genes upregulated by the SMAD3‐2KQ mutant were associated with inflammatory and immune response‐related signaling pathways. C) Hierarchical clustering analysis of patient gene expression data (TCGA) indicates that the 14‐gene signature resulted in two main clusters indicated in blue and red, respectively. Expression data of 17 TNBC patients with metastasis (+M) and recurrence (+R) and 144 TNBC patients without metastasis (−M) and recurrence (−R) were downloaded from TCGA. D) GSEA analyses showing that KAT6A‐acetylated SMAD3 upregulates IL‐6, IL‐22, and TNF*α* signaling pathways. NES, normalized enrichment score. E) qRT‐PCR analyses of upregulation of *IL‐6*, *IL‐22*, and *TNFA* by SMAD3‐2KQ mutant in MDA‐MB‐231/sgSMAD3 and BT‐549/sgSMAD3 cells without TGF‐*β*1 treatment. F) Enzyme linked immunosorbent assay (ELISA) of expression of IL‐6, IL‐22, and TNF*α* in the condition medium from cells in panel (E). G) In panels (E) and (F), data are representative of three independent experiments with similar results. Error bars, SEM. *P < 0.05, **P < 0.01, and ***P < 0.001 by paired two‐tail *t*‐test.

Given the established role of SMAD3 as a critical transcriptional factor,^[^ ^33^, ^34^ ^]^ we hypothesized that K20/K117 acetylation of SMAD3 enhances its transcriptional activity. To test this hypothesis, we performed RNA‐Seq analysis in MDA‐MB‐231/sgSMAD3 cells with re‐expression of sgRNA‐resistant Flag‐SMAD3 WT or 2KQ mutant without the TGF*β*1 treatment. We identified 554 genes whose expression were significantly upregulated by re‐expression of sgRNA‐resistant SMAD3‐2KQ mutant compared to SMAD3‐WT (fold change >2, *P* <0.05) (**Figure** **3**A). These 554 genes were highly enriched in gene ontologies that are associated with the inflammatory & immune response‐related signaling pathways (Figure 3B). We then used this signature to stratify publicly available gene expression data from TNBC patients (TCGA) and revealed that 14 genes were highly coexpressed in specimens of TNBC patients with metastasis and recurrence compared with those in no metastatic and recurrence TNBC patients (Figure 3C). Gene set enrichment analysis (GSEA) further revealed that the inflammatory and immune response‐related gene signatures including *IL‐6*, *IL‐22*, and *TNFA*, were significantly altered by re‐expression of SMAD3‐2KQ mutant (Figure 3D). In addition, we validated that the 2KQ mutant promoted the expression and secretion of IL‐6, IL‐22, and TNF*α* in MDA‐MB‐231/sg SMAD3 and BT‐549/sg SMAD3 cells (Figure 3E,F). We further performed *IL‐6*, *IL‐22*, and *TNFA* promoter luciferase activity analysis and found that compared to SMAD3‐WT, re‐expression of SMAD3‐2KQ mutant significantly increased the *IL‐6*, *IL‐22*, and *TNFA* promoter activity (Figure S4A, Supporting Information). Additionally, SMAD3‐2KQ mutant markedly enhanced the binding of SMAD3 to the promoters of *IL‐6*, *IL‐22*, and *TNFA* in MDA‐MB‐231 and BT‐549 cells by chromatin immunoprecipitation quantitative‐PCR (ChIP‐qPCR) analysis (Figure S4B–D, Supporting Information). Meanwhile, we also found that TGF‐*β*1 stimulation can indeed cause the increase of these cytokines expression in a KAT6A‐dependent manner (Figure S4E, Supporting Information). These data indicate that the K20/K117 acetylation of SMAD3 upregulates immune response‐related cytokines.

### 2.4 The K20/117 Acetylation Increases SMAD3 Association with KAT6A‐Mediated H3K23ac Reader TRIM24 and Thereby Enhances SMAD3 Activity

**Figure 4:**
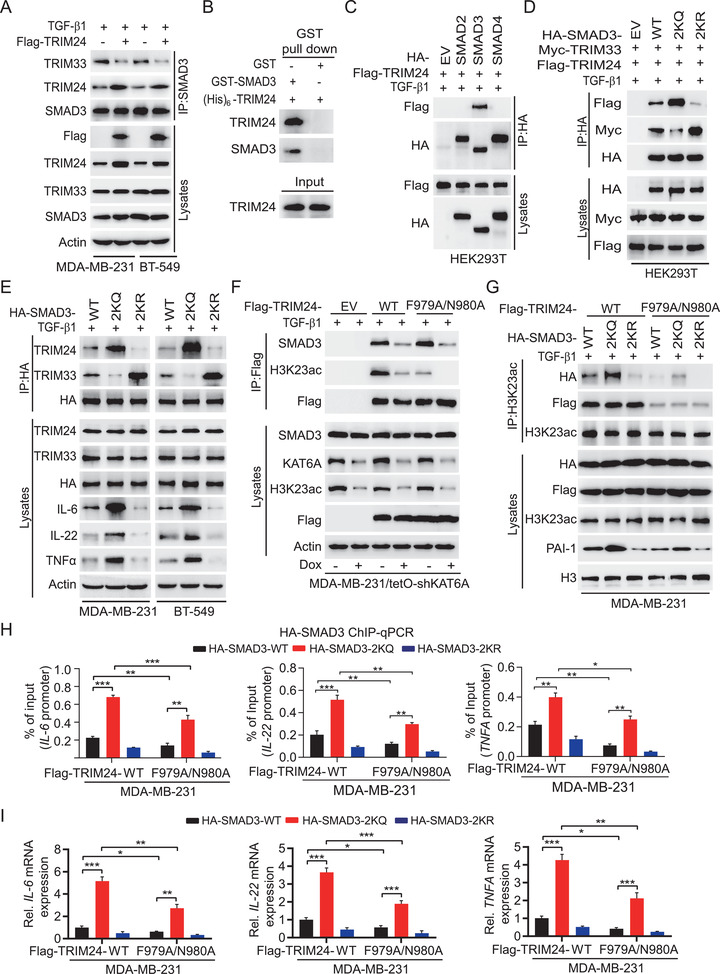
The K20/117 acetylation increases SMAD3 association with KAT6A‐mediated H3K23ac reader TRIM24 and thereby enhances SMAD3 activity. A) Oncogenic TRIM24 overexpression enhanced its association with SMAD3 and disrupted tumor suppressor TRIM33–SMAD3 interaction in MDA‐MB‐231 and BT‐549 cells stimulated with TGF‐*β*1. B) GST pull‐down assay was performed using purified GST‐SMAD3 and (His)_6_‐TRIM24, followed by WB with anti‐TRIM24 and anti‐SMAD3 antibodies. C) IP and WB of TRIM24 binding with SMAD2/3/4 in HEK293T cells stimulated by TGF‐*β*1. D) IP and WB of the effect of the K20/117Q (2KQ) or K20/117R (2KR) mutant on SMAD3 binding with TRIM24 and TRIM33 in HEK293T cells. E) IP and WB of the effects of the 2KQ or 2KR mutant on SMAD3 binding with TRIM24 and TRIM33, and IL‐6, IL‐22, and TNF*α* expression in MDA‐MB‐231 and BT‐549 cells. F) Effects of re‐expression of shRNA‐resistant Flag‐TRIM24 WT, F979A/N980A mutant, or an empty vector control (EV) on TRIM24 association with H3K23ac and SMAD3 in MDA‐MB‐231/tetO‐shKAT6A cells. G) Ectopic expression of TRIM24 F979A/N980A mutant decreases H3K23ac association with TRIM24 and SMAD3 in MDA‐MB‐231 cells. H) ChIP‐qPCR analyses of effects of ectopic expression of TRIM24 F979A/N980A mutant on SMAD3 binding to the promoters of *IL‐6*, *IL‐22*, and *TNFA* in MDA‐MB‐231 cells. I) Ectopic expression of TRIM24 F979A/N980A mutant decreased SMAD3‐mediated expression of *IL‐6*, *IL‐22*, and *TNFA*. Data are representative of three independent experiments with similar results. Error bars, SD. *P < 0.05, **P < 0.01, and ***P < 0.001 by paired two‐tail *t*‐test.

The biological functions of SMAD3 are modulated by its binding partners.^[^ ^33^, ^34^ ^]^ TRIM33 is a member of the tripartite motif (TRIM) protein family and is recruited to SMAD3, leading to transcription inhibition of SMAD3 targeted genes.^[^ ^35^ ^]^ TRIM24 is a TRIM33 related cofactor in TRIM superfamily^[^ ^36^ ^]^ and a reader protein of KAT6A‐mediated H3K23ac in cancers.^[^ ^10^, ^37^, ^38^ ^]^ Thus, we investigated whether the K20/117 acetylation impacts SMAD3 association with TRIM33 and/or TRIM24. As shown in **Figure** **4**A, both TRIM24 and TRIM33 bound to SMAD3 in MDA‐MB‐231 and BT‐549 cells with TGF‐*β*1 stimulation. However, overexpression of TRIM24 enhanced its association with SMAD3 and disrupted the association between TRIM33 and SMAD3 (Figure 4A). Then we performed a GST pull‐down assay with recombinant GST‐SMAD3 and (His)_6_‐TRIM24 and found that TRIM24 directly bound to SMAD3 (Figure 4B). SMAD3 but not SMAD2 and SMAD4 showed its specifically association with TRIM24 (Figure 4C). In addition, the C‐terminal MH2 domain (amino acids 232–425) but not the N‐terminal MH1 and Linker domain (1–231 amino acid) segment of SMAD3‐mediated SMAD3–TRIM24 association (Figure S5A, Supporting Information). We also generated several deletion mutants lacking various functional binding domains (Figure S5B, Supporting Information) and identified that the C‐terminal deletion mutant of TRIM24 (D1), but not the N‐terminal deletion mutant (D2), associated with SMAD3 (Figure S5C, Supporting Information). When D1 and four additional N‐terminal truncated mutants, D3 to D6, were separately coexpressed with WT TRIM24, none of these deletion mutants interacted with SMAD3 (Figure S5D, Supporting Information), suggesting that amino acid residues 393–823 in the middle segment of TRIM24 protein mediate its association with SMAD3. This was further validated by coexpression of SMAD3 with the truncated D7 mutant of TRIM24 (Figure S5E, Supporting Information). The activated MH2 domain in SMAD3 also specifically and selectively binds to the middle region of TRIM33 through direct interactions.^[^ ^39^ ^]^ Thus, forced overexpression of TRIM24 inhibited the formation of SMAD3–TRIM33 complex (Figure S5F, Supporting Information). Conversely, TRIM33 overexpression inhibited SMAD3–TRIM24 interaction (Figure S5G, Supporting Information). All these data suggest that TRIM24 and TRIM33 competitively share the pool of TGF*β*‐activated SMAD3.

Next, we investigated the effect of the K20/117 acetylation on SMAD3 association with TRIM24 and TRIM33. As shown in Figure 4D, compared with WT SMAD3, the acetylation‐mimetic 2KQ mutant enhanced TRIM24–SMAD3 interaction while inhibiting TRIM33 association with SMAD3. Conversely, the acetylation‐deficient 2KR mutant impaired TRIM24–SMAD3 interaction and promoted TRIM33 association with SMAD3 (Figure 4D). This observation was further validated in MDA‐MB‐231 and BT‐549 cells with overexpression of SMAD3 WT, 2KQ, or 2KR mutant (Figure 4E). In addition, ectopic expression of the 2KQ mutant but not the 2KR mutant increased protein expression of IL‐6, IL‐22, and TNF*α* compared to the WT (Figure 4E). These data demonstrate that the acetylation of SMAD3 promotes its interaction with oncogenic TRIM24 and reduces its association with TRIM33 tumor suppressor, leading to enhanced SMAD3 oncogenic activity.

Given that KAT6A‐acetylated H3K23 reader TRIM24 acts as an oncogenic transcriptional coactivator^[^ ^40^ ^]^ and high levels of H3K23ac correlated with poor prognosis of breast cancer,^[^ ^41^ ^]^ we tested a hypothesis that KAT6A‐acetylated H3K23 recruits TRIM24–SMAD3 complex and enhances SMAD3–chromatin interaction, resulting in enhanced SMAD3 activation in breast cancer. We generated a TRIM24 F979A/N980A mutant that disrupts TRIM24–H3K23ac interaction.^[^ ^38^ ^]^ Consistent with our previous report,^[^ ^10^ ^]^ in MDA‐MB‐231 tetO cells with a KAT6A shRNA, Dox‐induced *KAT6A* KD decreased H3K23 acetylation (H3K23ac) and the association with TRIM24 and H3K23ac compared with the noninduced control (Figure 4F). Compared to TRIM24 WT, the F979A/N980A mutation markedly attenuated the association between TRIM24 and H3K23ac but not TRIM24 association with SMAD3 (Figure 4F). In addition, we coexpressed HA‐tagged SMAD3 WT, 2KQ, or 2KR mutant with WT TRIM24 or the F979A/N980A mutant in MDA‐MB‐231 cells and found that compared to WT TRIM24, F979A/N980A mutation decreased H3K23ac association with TRIM24 as well as SMAD3 WT and mutants (Figure 4G). Compared to TRIM24 WT, F979A/N980A mutation decreased the binding of SMAD3 WT and 2KQ mutant to the promoter of *IL‐6*, *IL‐22*, and *TNFA*, respectively (Figure 4H), leading to inhibition of the expression of *IL‐6*, *IL‐22*, and *TNFA* (Figure 4I). In addition, compared to SMAD3 WT in F979A/N980A mutation group, the 2KQ mutation markedly increased its binding to the promoter of *IL‐6*, *IL‐22*, and *TNFA* (Figure 4H) and thereby moderately enhanced the expression of *IL‐6*, *IL‐22*, and *TNFA* (Figure 4I). This data suggests that SMAD3 2KQ mutation was also able to increase the expression of *IL‐6*, *IL‐22*, and *TNFA* without H3K23ac association and H3K23ac binding further increases SMAD3 2KQ mutant activity. Moreover, H3K23ac was demonstrated not to directly bind to SMAD3 2KQ mutant by a GST pull‐down assay (Figure S5H, Supporting Information). Taken together, these results demonstrate that KAT6A‐acetylated SMAD3 promotes SMAD3 binding to H3K23ac reader TRIM24 and KAT6A‐mediated H3K23ac further increases TRIM24/SMAD3–chromatin association, leading to enhanced SMAD3 signaling activation.

### 2.5 The K20/K117 Acetylation of SMAD3 Promotes TNBC Metastasis by Enhancing Breast Cancer Stem‐Like Cell (CSC) Properties and MDSCs Recruitment

**Figure 5:**
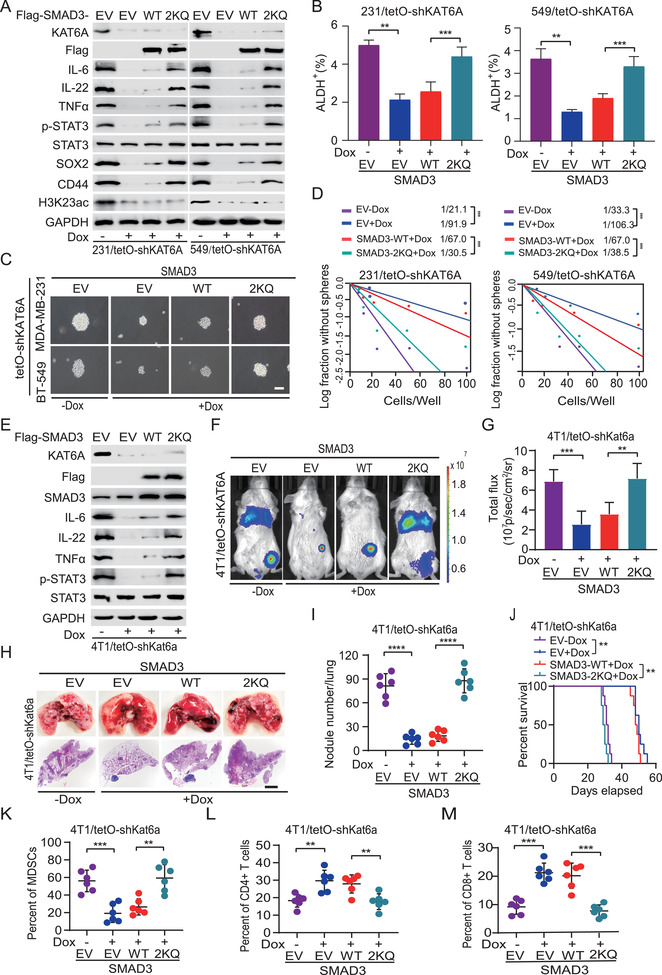
K20/K117 acetylation promotes metastasis by enhancing breast cancer stem‐like cell properties and MDSCs recruitment. A) Effects of ectopic expression of SMAD3 2KQ mutant or WT on H3K23ac, IL‐6, IL‐22, and TNF*α* expression, STAT3 phosphorylation (p‐STAT3), and breast cancer stem‐like cell marker expression of SOX2, and CD44 in MDA‐MB‐231/tetO‐shKAT6A and BT‐549/tetO‐shKAT6A cells. MDA‐MB‐231 and BT‐549 stable cells with a tetO‐shKAT6A were pretreated with or without Dox for 24 h and then stimulated with TGF‐*β*1 (5 ng mL^−1^, 2 h). EV, an empty vector control. B) ALDH^+^ populations were analyzed in MDA‐MB‐231 (231) and BT‐549 (549) cells from panel (A) by ALDEFLUOR assay. C) The representative mammosphere sphere images are shown. Scale bars, 100 µm. D) Limiting dilution mammosphere‐forming assays of effects of ectopic expression of SMAD WT or 2KQ mutant in MDA‐MB‐231/shKAT6A and BT‐549/shKAT6A cells. E) Effects of overexpression of SMAD WT or 2KQ mutant on IL‐6, IL‐22, and TNF*α* expression, and p‐STAT3 in 4T1/tetO‐shKAT6A cells. 4T1/tetO stable cells with a shKAT6A were pretreated with or without Dox for 24 h and then stimulated with TGF‐*β*1 (5 ng mL^−1^, 2 h). EV, an empty vector control. F) Representative bioluminescence (BLI) images of orthotopic implantation assays. The 4T1 cells expression indicated DNA constructs were inoculated into the mammary fat pad of BALB/c mice (*n* = 6). G) Quantification of BLI in panel (F). H) Representative bright‐field (upper panel) and H&E staining (lower panel) imaging of the lungs in panel (F). Scale bars: 100 µm. I) Quantification of the number of surface lung metastasis in panel (H). J) Kaplan–Meier survival analysis of animals with 4T1 tumor xenografts (*n* = 6). Flow cytometric analysis (FACS) of K) tumor‐associated MDSCs (CD45^+^CD11b^+^Gr1^+^), and L) CD4^+^ (CD45^+^CD3^+^CD4^+^) T cells and M) CD8^+^ (CD45^+^CD3^+^CD8^+^) T cells in the lungs from panel (H). Data are representative of three independent experiments with similar results. Error bars, SEM. **P < 0.01 and ***P < 0.001, by paired two‐tail *t*‐test or log‐rank analysis.

Myeloid cells derived from bone marrow contribute to the formation of the premetastatic microenvironment^[^ ^42^ ^]^ and persistent production of immune response‐related cytokines in tumor cells increases MDSCs recruitment, resulting in the promotion of CSC properties, cancer immune escape, and metastasis.^[^ ^7^, ^24^, ^43^ ^]^ Thus, we investigate whether upregulation of IL‐6, IL‐22, and TNF*α* by the KAT6A–SMAD3 axis described above is critical for breast cancer stem‐like cell (BCSC) properties in TNBC. Compared with that in the noninduced cells, Dox‐induced KD of *KAT6A* decreased IL‐6, IL‐22, and TNF*α* protein expression, STAT3 phosphorylation (p‐STAT3), the expression of CSC‐related proteins SOX2, and CD44 (**Figure** **5**A), ALDH^+^ subpopulations (Figure 5B), mammosphere sizes (Figure 5C), and mammosphere formation ability (Figure 5D) in MDA‐MB‐231 and BT‐549 cells. Ectopic expression of the acetylation‐mimetic SMAD3 2KQ mutant, but not WT, rescued *KAT6A* KD‐inhibited IL‐6, IL‐22, TNF*α* protein expression, p‐STAT3, the expression of CSC‐related proteins (Figure 5A), ALDH^+^ subpopulations (Figure 5B), mammosphere sizes (Figure 5C), and mammosphere formation ability (Figure 5D). In addition, compared with the control, *KAT6A* KD increased epithelial marker E‐cadherin expression and decreased the protein expression of mesenchymal markers N‐cadherin, ZEB1, Slug, and Snail in MDA‐MB‐231/sgSMAD3 cells with re‐expressing SMAD3 WT but not K20/117R mutant (Figure S6A, Supporting Information). These data support that KAT6A‐dependent SMAD3 acetylation is important for BCSC properties.

To investigate the functions of KAT6A‐dependent SMAD3 acetylation in modulation of TNBC metastasis, we first re‐expressed Flag‐SMAD3 WT and 2KR mutant in 4T1/sgSmad3 cells with or without *Kat6a* KD. As shown in Figure S6B in the Supporting Information, *Kat6a* KD significantly decreased the expression of IL‐6, IL‐22, TNF*α*, and p‐STAT3 in 4T1/sgSmad3 cells with re‐expressing SMAD3 WT but not K20/117R mutant. At 16 h, compared with WT SMAD3, *Kat6a* KD or re‐expression of SMAD3 2KR mutant did not impair cell proliferation (Figure S6C, Supporting Information). However, *Kat6a* KD sharply impaired the cell invasion, migration, and MDSCs recruitment in vitro in 4T1/sgSmad3 cells with re‐expressing SMAD3 WT but not K20/117R mutant (Figure S6D–F, Supporting Information). MDSCs recruitment was further validated by treatment with KAT6A inhibitor WM‐1119^[^ ^44^ ^]^ (Figure S6G, Supporting Information), anti‐IL‐6, anti‐IL‐22, or anti‐TNF*α* antibody neutralization of SMAD3‐induced cytokine IL‐6, IL‐22, or TNF*α*, respectively (Figure S6H, Supporting Information). In addition, we assessed micrometastases in lungs and employed a PKH26‐labeled MDSCs model to detect effects of SMAD3 acetylation on rapid recruitment of MDSCs in vivo. *Kat6a* KD decreased micrometastases in lungs and MDSC rapid recruitment (Figure S7A–D, Supporting Information). Ectopic expression of SMAD3 2KQ mutant rescued *Kat6a* KD‐inhibited micrometastases in lungs and MDSC rapid recruitment (Figure S7A–D, Supporting Information). These data support that KAT6A‐induced acetylation of SMAD3 promotes TNBC metastasis.

Next, we employed the mouse 4T1 murine mammary carcinoma metastasis model to determine the effects of SMAD3 acetylation on the recruitment of MDSCs in vivo. Compared with that in the noninduced cells, Dox‐induced KD of *KAT6A* decreased IL‐6, IL‐22, and TNF*α* protein expression, p‐STAT3 (Figure 5E). Ectopic expression of the acetylation‐mimetic SMAD3 2KQ mutant, but not WT, rescued *KAT6A* KD‐inhibited IL‐6, IL‐22, TNF*α* protein expression, and p‐STAT3 (Figure 5E). Consistent with previous observations, KD of *KAT6A* inhibited tumor growth (Figure 5F,G), lung metastasis (Figure 5H,I), and prolonged survival of orthotopic 4T1 tumor xenograft‐bearing animals (Figure 5J). We further revealed that *KAT6A* KD reduced MDSCs recruitment to the metastastic lung tissues (Figure 5K; Figure S7F,G, Supporting Information) and primary tumors (Figure S7H, Supporting Information), and CD4^+^/CD8^+^ T cell depletion in metastatic lung tissues (Figure 5L,M; Figure S7E,G, Supporting Information). Ectopic expression of the SMAD3 2KQ mutant, but not WT, not only rescued *KAT6A* KD‐inhibited tumor growth (Figure 5F,G), lung metastasis (Figure 5F,H,I), markedly reduced the animal survival (Figure 5J), but also promoted MDSCs recruitment to lung tissues (Figure 5K; Figure S7G, Supporting Information) and primary tumor (Figure S7H, Supporting Information), CD4^+^/CD8^+^ T cells depletion (Figure 5L,M; Figure S7G, Supporting Information) in metastatic lung tissues. In addition, we applied established three patient‐derived xenograft (PDX) models to determine the KAT6A/SMAD3 axis and found that KAT6A expression was highly consistent with the levels of SMAD3 K20/117ac and its downstream effectors IL‐6 expression and p‐STAT3 (Figure S7I, Supporting Information). Taken together, these results demonstrate that KAT6A‐dependent SMAD3 acetylation promotes TNBC metastasis through MDSCs recruitment.

To further demonstrate the critical role of the proinflammatory cytokine secretion by breast cancer cells in SMAD3‐mediated tumor metastasis, we generated *IL‐6* KO 4T1 cells (Figure S8A, Supporting Information) and found that *IL‐6* KO inhibited SMAD3‐2KQ‐induced MDSCs recruitment (Figure S8B,C, Supporting Information). We injected the cells into the *IL6* ^−/−^ mice through tail vein injection to prevent confounding effects from the host IL‐6 and determined the impact of *IL‐6* KO in SMAD3‐mediated tumor metastasis (Figure S8D, Supporting Information). Consistent with the above observations, ectopic expression of the SMAD3‐2KQ mutant promoted tumor growth (Figure S8E,F, Supporting Information), lung metastasis (Figure S8G, Supporting Information), reduced survival of 4T1 tumor xenograft bearing animals (Figure S8H, Supporting Information), and promoted MDSCs accumulation (Figure S8I, Supporting Information), CD4^+^ and CD8^+^ T cell depletion (Figure S8J,K, Supporting Information) whereas it did not affect macrophage recruitment and the infiltration of B cells and NK cells (Figure S8L–N, Supporting Information). However, *IL6* KO inhibited tumor growth, lung metastasis, extended survival of animals bearing‐tumor xenografts (Figure S8E–H, Supporting Information), reduced MDSC recruitment, CD4^+^ and CD8^+^ T cell depletion (Figure S8I–K, Supporting Information), macrophage repression (Figure S8L, Supporting Information), and had no effect on the infiltration of B cells and NK cells (Figure S8M,N, Supporting Information). Taken together, these data demonstrate that KAT6A‐induced acetylation of SMAD3 promoted metastasis not only by enhancing breast cancer stem‐like cell properties but also by inducing the MDSCs recruitment by persistent production of immune response‐related cytokines.

### 2.6 Targeting KAT6A Sensitizes PD‐L1 Immunotherapy in TNBC by Decreasing MDSCs Recruitment

**Figure 6:**
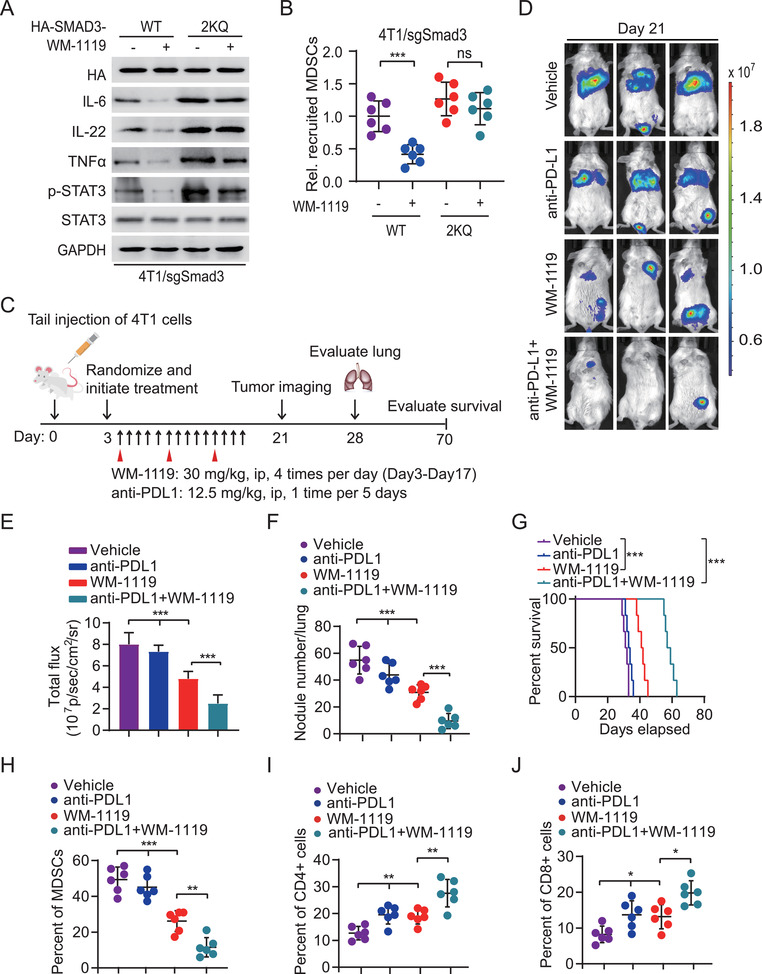
Targeting KAT6A sensitizes PD‐L1 immunotherapy in TNBC by decreasing MDSCs recruitment. A) Effects of treatment of KAT6A inhibitor WM‐1119 on SMAD3 acetylation and IL‐6, IL‐22, TNF*α*, and p‐STAT3 expression in 4T1 cells with a SMAD3 sgRNA. 4T1 cells were transduced with sgRNA resistant WT SMAD3 or the 2KQ mutant. B) Transwell migration analysis of MDSCs recruitment by indicated 4T1 cells treated with or without WM‐1119. C) Treatment scheme for the evaluation of in vivo efficacy of WM1119 in combination with an anti‐PD‐L1 antibody in 4T1 tumor xenografts. The mice were treated with indicated 30 mg kg^−1^ WM1119 with or without 12.5 mg kg^−1^ of the anti‐PD‐L1 antibody within 2 weeks following the indicated administration time and frequency. D) Representative BLI images of treated mice on day 21. E) Quantification of the BLI activity in panel (D). F) Quantification of the number of surface lung metastasis in panel (D). G) Kaplan–Meier survival analysis of animals with indicated 4T1 tumors (*n* = 6). FACS of H) recruited MDSCs, and I) CD4^+^ T cells and J) CD8^+^ T cells in metastatic lung tissues. Data are representative of two or three independent experiments with similar results. Error bars, SEM. *P < 0.05, **P < 0.01, and ***P < 0.001 by paired two‐way Student's *t*‐test, one‐way ANOVA, or log‐rank analysis.

Although targeting immune checkpoints such as programmed cell death protein 1 (PD‐1) and its ligand, programmed cell death ligand 1 (PD‐L1), or cytotoxic T lymphocyte‐associated antigen‐4 (CTLA‐4) has achieved noteworthy benefit in multiple cancers by blocking immunoinhibitory signals and enabling effective antitumor responses,^[^ ^45^, ^46^ ^]^ limited responses to these immunotherapies were reported in patients with metastatic breast cancer, emphasizing the need to identify strategies that will increase response efficacy. Thus, we investigated whether targeting KAT6A by using an inhibitor WM‐1119 will improve the efficacy of PD‐L1 immunotherapy in treating breast cancer. We first assessed whether the KAT6A inhibitor WM‐1119 can inhibit SMAD3 2KQ mutant‐upregulated IL‐6, IL‐22, and TNF*α* protein expression and MDSCs recruitment in vitro in 4T1 cells. As shown in **Figure** **6**A,B, treatment with WM‐1119 reduced the levels of acetylation of SMAD3 K20 and K117, the expression of IL‐6, IL‐22, and TNF*α*, p‐STAT3, and MDSCs recruitment in 4T1/sgSMAD3 cells re‐expressing WT SMAD3 but not the 2KQ mutant.

Next, 4T1 cells with stable expression of luciferase were transplanted into immunocompetent mice through tail veins. After randomization by bioluminescence imaging (BLI) flux values to ensure that tumors were of equal size in the treatment and control groups, animals were initially treated with an anti‐PD‐L1 antibody, WM‐1119, or a combination of them (Figure 6C). Similar as previous reports,^[^ ^47^, ^48^ ^]^ compared with the vehicle cohort, single treatment with the anti‐PD‐L1 antibody had no appreciable effects on tumor burden (Figure 6D,E), metastasis lung nodule number (Figure 6F), and animal survival (Figure 6G). A significant reduction of tumor burden and metastasis lung nodule number was observed in tumor‐bearing mice with the WM‐1119 treatment (Figure 6D–F). Significant improvement in animal survival of the WM‐1119‐treated cohort compared with the vehicle and the anti‐PD‐L1 antibody‐treated cohorts (*P* < 0.001) (Figure 6G). Combination treatment of the anti‐PD‐L1 antibody and WM‐1119 further inhibited tumor burden and lung metastasis (Figure 6D–F). Moreover, significant improvement in animal survival of the combined‐treated cohort compared with the WM‐1119 treated cohort (*P* < 0.001) (Figure 6G). Consistent with these, treatment with WM‐1119 but not the anti‐PD‐L1 antibody significantly decreased MDSCs recruitment (Figure 6H) and CD4^+^/CD8^+^ T cell depletion (Figure 6I,J). The combination treatment further inhibited MDSCs recruitment and CD4^+^/CD8^+^ T cell depletion compared with the WM‐1119‐treated cohort (Figure 6H–J). These results improved the effects of the PD‐L1 immunotherapy and the combination treatment could be beneficial to breast cancer patients with metastasis.

### 2.7 Correlative Expressions of KAT6A, Acetylated SMAD3, and CD11b Are Prognostic for Clinical TNBC

**Figure 7:**
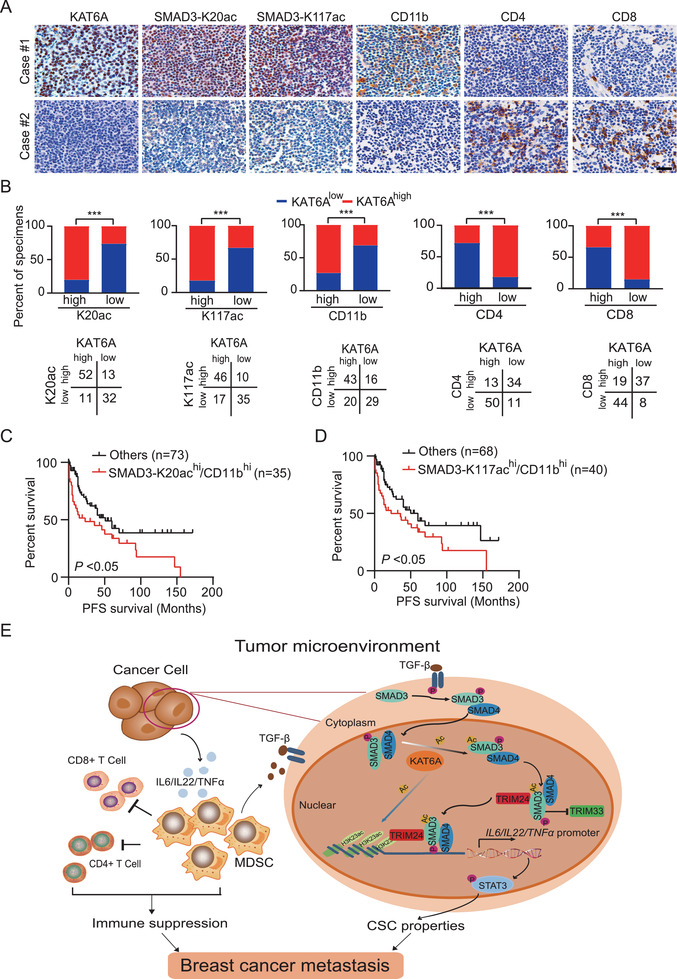
Correlative expressions of KAT6A, acetylated SMAD3, and CD11b are prognostic for clinical TNBC. A) Representative images of IHC staining for KAT6A, SMAD‐K20ac, SMAD‐K117ac, MDSCs marker CD11b, and T cell markers CD4 and CD8 in metastatic lymph nodes from clinical breast cancer specimens. Data are representative of two independent experiments. Scale bars, 50 µm. B) Correlation of expression between KAT6A, SMAD3‐K20ac, SMAD3‐K17a, CD11b, CD4, and CD8 in panel (A). ***P < 0.001 by chi‐square test. Kaplan–Meier analyses of the progression‐free survival for TNBC patients with high C) SMAD‐K20ac or D) SMAD‐K117ac and high CD11b. *P < 0.05 by log‐rank analysis. E) A working model of KAT6A acetylation of SMAD3 signaling in TNBC metastasis. KAT6A acetylates SMAD3 at K20 and K117, which promotes SMAD3 association with TRIM24 and disrupts TRIM33–SMAD3 interaction. KAT6A‐acetylated H3K23 further recruits and stabilizes TRIM24–SMAD3 interaction with chromatin, leading to transcriptional upregulation of *IL‐6*, *IL‐22*, and *TNFA*. Thus, SMAD3 acetylation not only promotes CSC phenotype of tumor cells, but also enhances MDSCs recruitment to enhance immune inhibition. In addition, MDSC‐secreted TGF*β* reactivates KAT6A/SMAD3 signaling, thereby forming a novel positive feedback loop between cancer cells and MDSCs to promote TNBC metastasis. Targeting KAT6A with a small molecular inhibitor WM‐1119 sensitizes PD‐L1 immunotherapy in cancer metastasis.

KAT6A overexpression and MDSCs recruitment are closely associated with a poor prognosis for patients with breast cancer.^[^ ^7^, ^15^, ^20^, ^49^ ^]^ To further determine the clinical relevance of our findings in this study, we examined expression of KAT6A, SMAD3‐K20ac, SMAD3‐K117ac, MDSCs marker CD11b, T cell markers CD4 and CD8 in metastatic lymph nodes from 108 clinical TNBC specimens using IHC analysis. As shown in **Figure** **7**A, the expression of SMAD3‐K20ac and SMAD3‐K117ac was positively correlated with expression of KAT6A and CD11b and was negatively correlated with CD4 and CD8 expression. Quantification of the IHC staining also indicated that these correlations were statistically significant (Figure 7B). Kaplan–Meier analyses of survival showed that a concomitant increase in expression levels of SMAD3‐K20ac or SMAD3‐K117ac and MDSCs numbers was correlated with a shorter progression‐free survival in TNBC patients with metastasis (Figure 7C,D).

## 3 Discussion

TNBC is a life‐threating disease because of limited therapies and lack of effective therapeutic targets. While accumulated data demonstrate that MDSCs within the TME play an essential role in breast cancer progression and metastasis, the underlying mechanism that tumor‐associated MDSCs in driving the aggressive nature and metastasis of TNBC is still unclear. In this study, we describe a novel KAT6A–SMAD3 signaling that regulates MDSCs recruitment, metastasis, and immunotherapy in TNBC. KAT6A directly binds to and acetylates SMAD3 at K20 and K117, which promotes SMAD3 association with oncogenic chromatin modifier TRIM24 and disrupts its interaction with tumor suppressor TRIM33. Then this event in turn promotes KAT6A acetylated H3K23‐mediated recruitment and further stabilization of TRIM24–SMAD3 complex with chromatin and thereby further enhances SMAD3 activation and immune response‐related cytokine upregulation, leading to enhanced MDSCs recruitment and TNBC metastasis through immune suppression (Figure 7E). These immune response‐related cytokine‐promoted CSC properties via STAT3 signaling and MDSC‐secreted TGF*β* ^[^ ^50^ ^]^ may further activate KAT6A/SMAD3 signaling to form a positive feedback loop to promote TNBC metastasis (Figure 7E). In addition, targeting KAT6A using WM‐1119 inhibitor markedly improved the efficiency of the PD‐L1 immunotherapy using an orthotopic xenograft model of breast cancer metastasis. A concomitant increase in expression levels of KAT6A, acetylated SMAD3, and MDSC numbers is a prognosis factor for TNBC patients with metastasis.

This study demonstrates that epigenetic regulator KAT6A plays a critical role in TNBC metastasis. Early studies have revealed that KAT6A gene fusions resulting from chromosome translocation causes preleukemia stem cell transformation.^[^ ^16^, ^17^, ^18^ ^]^ Increasing data demonstrate that KAT6A also regulates tumor progression.^[^ ^10^, ^14^ ^]^ KAT6A amplification and/or overexpression in luminal subtype breast cancers promoted tumor growth.^[^ ^15^, ^20^ ^]^ However, the mechanism by which KAT6A drives breast cancer progression is still elusive. Here, we demonstrate that KAT6A amplifies in TNBC subtype breast cancer and its amplification associates with TNBC prognosis. Amplification and/or overexpression of KAT6A abnormally activate SMAD3 signaling and promote TNBC metastasis through immunosuppression. A small‐molecular inhibitor targeting KAT6A increases the efficacy of anti‐PD‐L1 therapy by inhibiting TNBC metastasis and significantly prolongs the survival for tumor xenograft‐bearing animals. In addition, elevated KAT6A and acetylated SMAD3 associate with clinical TNBC metastasis and prognosis.

This study identified SMAD3 as a new KAT6A nonhistone substrate. KAT6A acetylation of the histone and nonhistone proteins plays a crucial role in regulating fundamental biological processes. KAT6A acetylated H3K23, H3K9, and H3K14 are important for tumorigenesis,^[^ ^10^, ^20^ ^]^ gene expression,^[^ ^51^, ^52^, ^53^ ^]^ and immune responses.^[^ ^11^ ^]^ Nonhistone protein p53 acetylated by KAT6A was identified in cell senescence.^[^ ^8^ ^]^ Here, we report that KAT6A as the authentic acetyltransferase for acetylation of SMAD3 K20/117 that is critical in recruiting MDSCs and enhancing metastasis. SMAD3 acetylation was implicated in pulmonary fibrosis, tumor progression, and gene expression.^[^ ^27^, ^28^, ^54^ ^]^ The present study not only corroborates KAT6A acetylated nonhistone protein SMAD3 but also presents additional findings on the role of acetylated SMAD3 in MDSCs recruitment and cancer metastasis.^[^ ^27^, ^28^, ^54^ ^]^ In addition, we identify only KAT6A, but not other KATs, as an acetyltransferase for SMAD3. Moreover, KAT6A directly acetylates SMAD3 at both K20 and K117, but not the other acetylation sites previously described.^[^ ^27^, ^28^ ^]^ Finally, SMAD3 acetylation at K20 and K117 is dependent on TGF‐*β*‐induced phosphorylation of SMAD3, which is consistent with SMAD3 nuclear translocation and the transcriptional activation of downstream target genes.^[^ ^55^ ^]^ The K20/117 acetylation of SMAD3 may impact its structure and thus enhances the interaction between KAT6A and SMAD3 as demonstrated that the KAT6A interaction with p53 was enhanced by p53 phosphorylation in MCF‐7 cells.^[^ ^56^ ^]^

MDSCs is a critical component of the TME and exerts the protumorigenic effect by suppressing T cell functions and promoting tumor metastasis.^[^ ^5^, ^6^, ^57^ ^]^ Our studies with human and mouse TNBC cells demonstrate that under TGF*β* stimulation, KAT6A acetylation of SMAD3 increases the recruitment of MDSCs and promotes metastasis by transcriptionally activating cytokines, which is consistent with prior studies that proinflammatory cytokines promote recruitment and aggregation of MDSCs into tumor tissue.^[^ ^23^, ^43^, ^58^, ^59^ ^]^ Our data demonstrated that KAT6A‐dependent the acetylation of SMAD by nuclear KAT6A depends on TGF‐*β*1‐induced phosphorylation of SMAD3, which promotes SMAD3 nuclear accumulation. TGF*β* is also produced by MDSCs for inhibiting the antitumor immunity, resulting in upregulated tumor burden.^[^ ^60^ ^]^ Thus, the KAT6A/SMAD3 signaling and tumor‐derived MDSCs may form a positive feedback regulatory network, resulting in aggressive TNBC properties.

In this study, we also demonstrated that KAT6A acetylation of SMAD3 not only regulates gene expression but also affects SMAD3 association with different co‐regulators. In normal cells, SMAD3 activity is inhibited by TRIM33, which ubiquitinates SMAD4, resulting in the disassembly of the SMADs transcriptional complex and a forced exit of SMAD2/3 from the nucleus.^[^ ^35^, ^61^ ^]^ In cancer, forkhead box M1 interacts with SMAD3 to sustain activation of the SMAD3/SMAD4 complex in the nucleus through preventing TRIM33 from binding SMAD3 and ubiquitinating SMAD4.^[^ ^62^ ^]^ Here, we show an additional mechanism by which TRIM24 activates SMAD3 transcriptional activity by its association with SMAD3 while disrupting TRIM33–SMAD3 interaction. Moreover, KAT6A acetylated H3K23 recruits TRIM24–SMAD3 complex, which promotes SMAD3–chromatin interaction and further enhances SMAD3 transcriptional activity.

Clinical outcomes of immune therapies are strongly influenced by the immune microenvironment. Antitumor immunity involves the complex interplay among immune, cancer, and cancer‐associated cells.^[^ ^63^, ^64^ ^]^ Specific DNA‐ and histone‐modifying enzymes contribute to both the immunogenicity of cancer cells and the lineage commitment and/or maturation of immune cells.^[^ ^65^, ^66^, ^67^ ^]^ Moreover, resistance to immune‐checkpoint inhibitors can be reverted by epigenetic manipulations in preclinical models.^[^ ^64^, ^68^ ^]^ Thus, epigenetic drugs in combination with PD‐1 or PD‐L1 antibody can potentially improve therapy efficacy. Our results suggest that the KAT6A inhibitor WM‐1119 as a potential epigenetic drug for combination therapy with a PD‐L1 antibody in treating TNBC patients with metastasis. This new finding of the KAT6A inhibitor warrants further investigation

## 4 Conclusion

Overall, our study reveals a novel signaling in modulating TNBC metastasis and the TME and demonstrates that targeting epigenetic factor KAT6A may be a potential option to enhance immunotherapy efficacy to treat metastatic TNBC. This finding also extends the repertoire of functions for SMAD3 and advances our current understanding of the role of SMAD3 in the cancer metastasis. SMAD3 functions as a transcriptional activator for inducing cytokine expression in the tumor microenvironment as well as an immunity repressor for enhancing MDSCs recruitment in driving cancer metastasis.

## 5 Experimental Section

### Patient Specimens

Paraffin‐embedded sections of breast cancer metastatic lymph nodes were collected from Ren Ji Hospital. The study protocol was approved by the Clinical Care and Use Committee of Ren Ji Hospital (Shanghai, China). Written informed consent was obtained from all participants in the study. These specimens were examined and diagnosed by independent pathologists. Primary breast tumor tissues used to generate PDXs were obtained from biopsy material of patients with TNBC after they provided written informed consent, and implanted as tumor fragments into cleared inguinal mammary fat pads. All the research was performed according to the provisions of the Declaration of Helsinki of 1975.

### Xenograft Studies

Pathogen‐free female BALB/c and athymic nude mice at 4–6 weeks of age were purchased from SLAC, Shanghai, China. The IL6 knock‐out mutant mice CByJ.129S2(B6)‐IL6^tm1Kopf^/J (Stock No: 007078) and matched control mice BALB/cByJ (Stock No: 001026) were purchased from the Jackson Laboratory. All animal experiments were conducted under the Institutional Animal Care and Use Committee (IACUC)‐approved protocols at Shanghai Jiao Tong University in accordance with NIH and institutional guidelines and the details are described in the Experimental Section in the Supporting Information.

### Cell Lines and Cell Culture

MCF10A, MCF‐7, SUM185, T47D, BT‐474, ZR7530, BT‐549, HCC1806, MDA‐MB‐453, MDA‐MB‐468, MDA‐MB‐231, HEK‐293T, and MEF cells (from the Chinese National Infrastructure of Cell Line Resource, Beijing, China) and immortalized *Kat6a^−/−^* MEFs were cultured in Dulbecco's Modified Eagle Medium (DMEM) supplemented with 10% fetal bovine serum (FBS) and 1% penicillin and streptomycin. 4T1 cells (ATCC) were maintained in RPMI‐1640 medium supplemented with 10% FBS and 1% penicillin and streptomycin. All the breast cancer cell lines were authenticated using STR DNA fingerprinting at Shanghai Biowing Applied Biotechnology Co., Ltd. (Shanghai, China). Mycoplasma contamination was detected by the LookOut Mycoplasma PCR Detection Kit (Sigma‐Aldrich).

### In Vitro Acetylation Assay

For in vitro acetylation analysis, 500 ng purified recombinant 6xHis‐tagged SMAD3 WT, K20R, or K117R mutant was separately incubated with 100 ng recombinant KAT6A/MOZ (488‐778) protein (Cat. No. 81923, Active Motif) in a 20 µL reaction buffer (50 × 10^−3^ m Tris‐HCl pH 8.6, 2 × 10^−3^ m MgCl_2_, and 1 × 10^−3^ m TCEP, 0.02% Triton X‐100) with 20 × 10^−6^ m acetyl‐CoA for 2 h at room temperature. The reaction products were detected by WB using an anti‐SMAD3 K20ac or anti‐K117ac antibody.

### MDSCs Isolation

Spleen of 4T1 tumor‐bearing BALB/c mouse was disrupted in PBS containing 2% FBS. Aggregates and debris were removed by passing cell suspension through a 70 µm mesh nylon strainer (STEMCELL Technologies, Catalog #27215). Centrifugation was carried out at 300 × *g* for 10 min and cells were resuspended at 1 × 10^8^ nucleated cells mL^−1^ in recommended medium. Mouse MDSCs (CD11b^+^Gr1^+^) was isolated by an EasySep Mouse MDSC (CD11b^+^Gr1^+^) Isolation Kit (STEMCELL Technologies, Catalog #19867) following the manufacturer's recommendation. For purity assessment of MDSCs by flow cytometry, the following fluorochrome‐conjugated antibodies were used: anti‐Mouse CD45 (30‐F11), anti‐Mouse CD11b (M1/70), and anti‐Mouse Gr1 (RB6‐8C5) antibodies (STEMCELL Technologies).

### Flow Cytometry Analysis (FACS) and Sorting

Single‐cell suspensions were prepared and incubated on the ice with a combination of antibodies for 30 min in the dark. FACS analysis was performed using the LSRII Flow Cytometer (BD Biosciences), and data were analyzed using the FlowJo software (Tree Star Inc.). For FACS sorting, Aria II or FACS Jazz instruments were used.

### Statistics

All Co‐immunoprecipitation (Co‐IP) and immunostaining were repeated three times with similar results detected. All statistical analyses were carried out by GraphPad Prism version 8.3 (GraphPad Software Inc., San Diego, CA, USA). The significance of the data between experimental groups was determined by one‐way analysis of variance (ANOVA) with Newman–Keuls post‐test or unpaired two‐tailed Student’ s *t*‐test. Survival analysis was calculated using the log‐rank test and the Kaplan–Meier method. A *P*‐value <0.05 represented a statistical significant difference. No statistical method was utilized to predetermine the sample size.

## Conflict of Interest

The authors declare no conflict of interest.

## Autho Contributions

B.Y. and F.L. contributed equally to this work. H.F. and Y.L. designed and supervised the project. B.Y., F.L., B.S., W.L., Q.S., and F.L. performed the experiments. B.Y., F.L., Y.L., and H.F. interpreted and/or reviewed the data. B.Y., F.L., S.‐Y.C., C.C., G.C., Y.L., and H.F. wrote or edited the manuscript. All of the coauthors reviewed the manuscript.

## Supporting information

Supporting Information

## Data Availability

RNA‐Seq data reported in this study have been deposited with the Gene Expression Omnibus under the accession GEO ID: GSE171230 (https://www.ncbi.nlm.nih.gov/geo/query/acc.cgi?acc=GSE171230). The data supporting the finding of this study are available within the article and its Supporting Information files or available from the corresponding author on reasonable request.
