# KAT6A Acetylation of SMAD3 Regulates Myeloid‐Derived Suppressor Cell Recruitment, Metastasis, and Immunotherapy in Triple‐Negative Breast Cancer

**DOI:** 10.1002/advs.202105793

**Published:** 2022-01-25

**Authors:** Bo Yu, Fei Luo, Bowen Sun, Wenxue Liu, Qiqi Shi, Shi‐Yuan Cheng, Ceshi Chen, Guoqiang Chen, Yanxin Li, Haizhong Feng

*Adv. Sci*. **2021**, *8*, 2100014

DOI: 10.1002/advs.202100014

In the originally published article, the representative BLI images of treated mice in **Figure** **6**D present the wrong samples (the third in the second row, and the third in the fourth row). The correct Figure 6D is shown below. The authors declare that this correction does not affect the description, interpretation, or the original conclusions of the manuscript. The authors regret the inconvenience this error may have caused.

**Figure 6:**
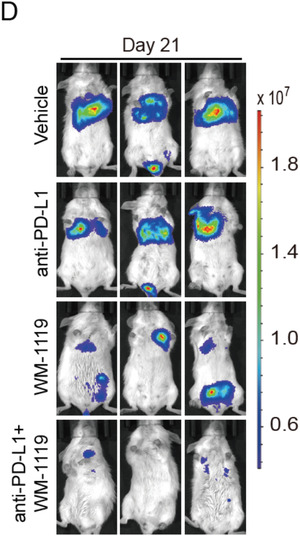
Targeting KAT6A sensitizes PD‐L1 immunotherapy in TNBC by decreasing MDSCs recruitment. D) Representative BLI images of treated mice on Day 21.

