# Supplementary material for: KAT6A Acetylation of SMAD3 Regulates Myeloid‐Derived Suppressor Cell Recruitment, Metastasis, and Immunotherapy in Triple‐Negative Breast Cancer
Source: Adv Sci (Weinh). 2021 Aug 13;8(20):2100014. doi: 10.1002/advs.202100014 (PMC8529494; doi:10.1002/advs.202100014)
Supplement: Supplementary file 1 — Supporting Information [file ADVS-8-2100014-s001.pdf]

© 2021 Wiley-VCH GmbH

ADVANCED SCIENCE
Open Access

Supporting Information

for *Adv. Sci.,* DOI: 10.1002/advs.202100014

KAT6A acetylation of SMAD3 regulates myeloid-derived suppressor cell recruitment, metastasis, and immunotherapy in triple-negative breast cancer

Bo Yu, Fei Luo, Bowen Sun, Wenxue Liu, Qiqi Shi, Shi-Yuan Cheng, Ceshi Chen, Guoqiang Chen, Yanxin Li, and Haizhong Feng

Supplementary information for

KAT6A acetylation of SMAD3 regulates myeloid-derived suppressor cell recruitment, metastasis, and immunotherapy in triple-negative breast cancer

Bo Yu, Fei Luo, Bowen Sun, Wenxue Liu, Qiqi Shi, Shi-Yuan Cheng, Ceshi Chen, Guoqiang Chen, Yanxin Li, and Haizhong Feng

Table S1
Figure S1-S8
Materials and Methods

Supplementary Table 1 MS identification of KAT6A binding with SMAD3 protein

peptide_calculate_ molecular weight	peptide_delta	peptide_score	peptide_expect	peptide_seq
897.4742	0.0032	45.07	0.0004	IPPGCNLK
1241.67	0.0032	66.91	7.70E-06	KGLPHVIYCR
1187.633	0.0025	57.39	6.40E-05	VLTQMGSPSIR
1291.671	0.0022	63.26	1.50E-05	FCLGLLSNVNR
1414.855	0.003	62.57	2.00E-06	VETPVLPPVLVPR
1425.69	0.0023	66.6	4.80E-06	WPDLHSHHELR
872.4716	0.002	55.43	9.90E-05	NAAVELTRR
1489.64	0.0043	62.47	3.20E-06	AMELCEFAFNMK
2102.086	0.0078	79.48	4.40E-07	TGQLDELEKAITTQNVNTK
3070.551	0.0121	73.57	1.60E-06	IFNNQEFAALLAQSVNQGFEAVYQLTR

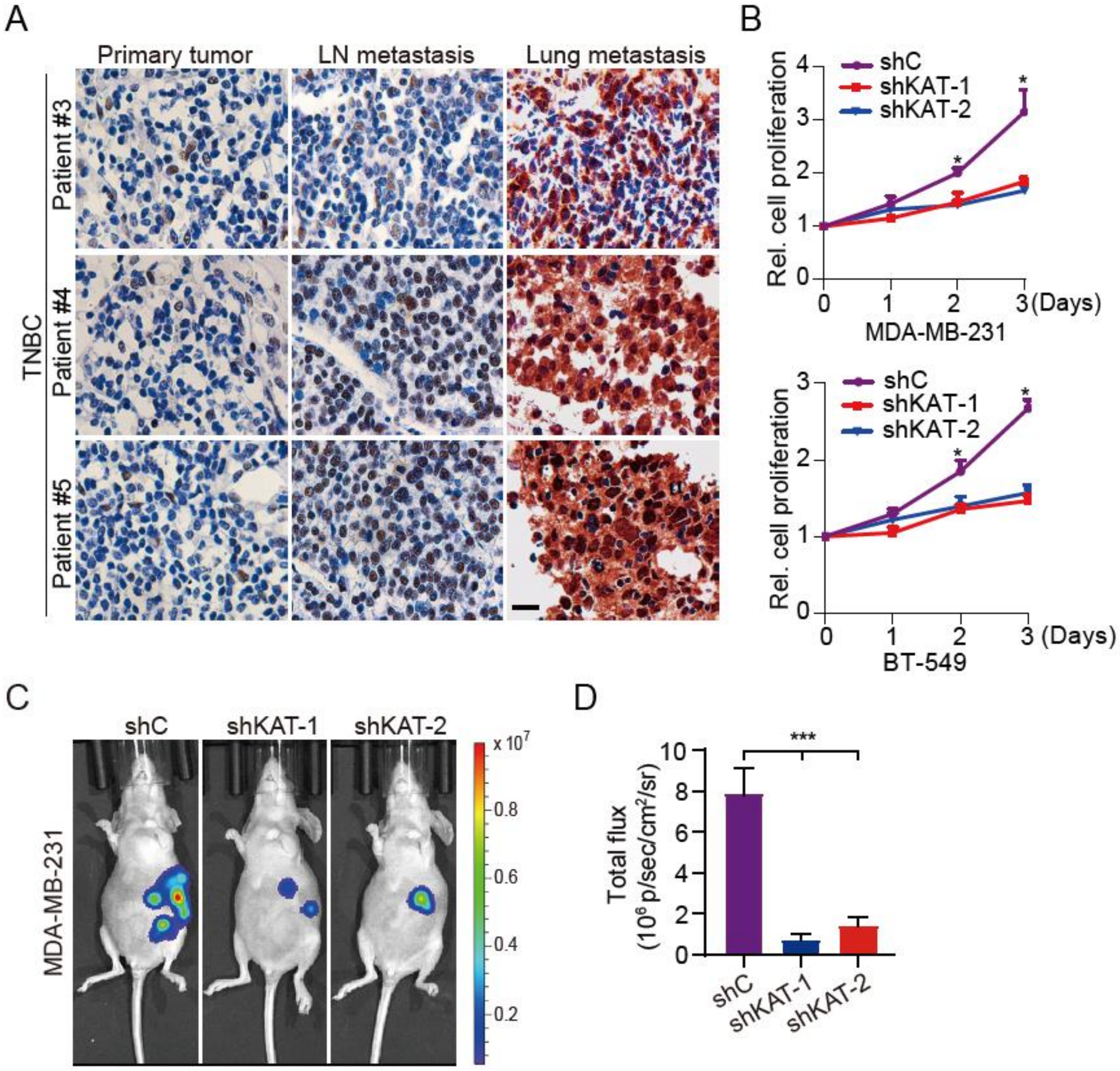

Supplementary Figure 1 KAT6A promotes TNBC cell proliferation in vitro and tumor growth in vivo. **A**, corresponding to Figure 1F, representative IHC staining of KAT6A protein in other three primary tumors and paired lymph nodes (LN) as well as lung metastases. Scale bars: 50 μm. **B**, Effects of *KAT6A* depletion on TNBC cell proliferation. **C**, Representative bioluminescence (BLI) images of MDA-MB-231/shC or MDA-MB-231/shKAT6A xenografts when injected into nude mouse gland fat pads (n = 6 per group). Mice were imaged at 4-5 weeks after implantation. **D**, Quantification of the BLI activity in **C**. Data are representative of three independent experiments with similar results. Error bars, SEM. *P < 0.05, ***P < 0.001, by one-way ANOVA.

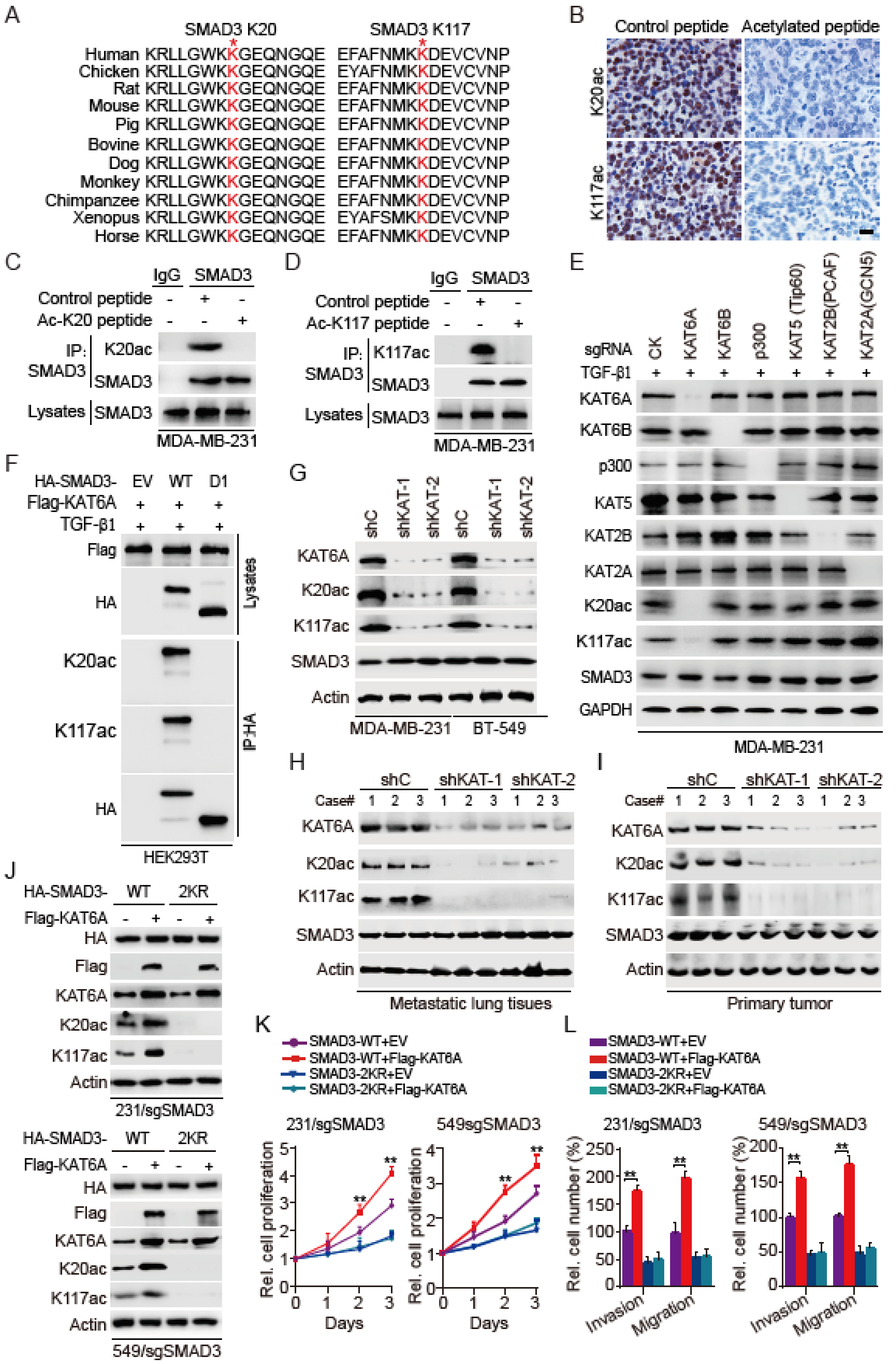

Supplementary Figure 2 Validating the specificity of the anti-SMAD3 K117 and K20 acetylation antibodies and identifying the importance of MH2 domain (D2 mutant) of

SMAD3 for SMAD3 acetylation by KAT6A. **A,** The amino acid sequences around K20 and K117 in SMAD3 among multiple species. **B,** IHC assays of a clinical breast cancer tumor tissue with the specific anti-K20ac or anti-117ac antibody in the presence of a control peptide or the specific acetylated peptide containing K20ac or K117ac. IHC was performed two times on the sample with the blocking peptide with similar results. Scale bar, 50 μm. **C** and **D**, IP and WB of K20ac (**C**) or K117ac (**D**) in MDA-MB-231 cells. A rabbit K20ac or K117ac was generated against a specific acetylated peptide containing K20ac or K117ac. Before IP, agarose beads were pre-incubated with a control peptide or the specific acetylated peptide containing K20ac or K117ac. **E**, WB of SMAD3-K20ac and -K117ac in MDA-MB-231 cells with various KAT sgRNAs or a control sgRNA (CK). **F**, The effect of the MH2 domain on SMAD3 acetylation by KAT6A. IP and WB of K20ac and K117ac in HEK293T cells with TGF-β1 stimulation. **G,** WB of effects of *KAT6A* depletion on SMAD3 K20ac and K117ac levels in MDA-MB-231 and BT-549 cells using two different KAT6A shRNAs (shKAT-1 and shKAT-2) or a control shRNA (shC). **H** and **I,** WB of effects of *KAT6A* depletion on SMAD3 K20ac and K117ac levels in metastatic lung tissues (**H**, from Figure 1K) and primary tumors (**I**, from Supplemental Figure S1C). **J-L**, Effect of KAT6A overexpression on SMAD3 acetylation (**J**), cell proliferation (**K**), invasion and migration (**L**) in MDA-MB-231/sgSMAD3 and BT-549/sgSMAD3 cells. MDA-MB-231/sgSMAD3 and BT-549/sgSMAD3 stable cells were co-transfected with Flag-KAT6A and sgRNA resistant-HA-SMAD3 WT or 2KR mutant. Data are representative of two independent experiments with similar results.

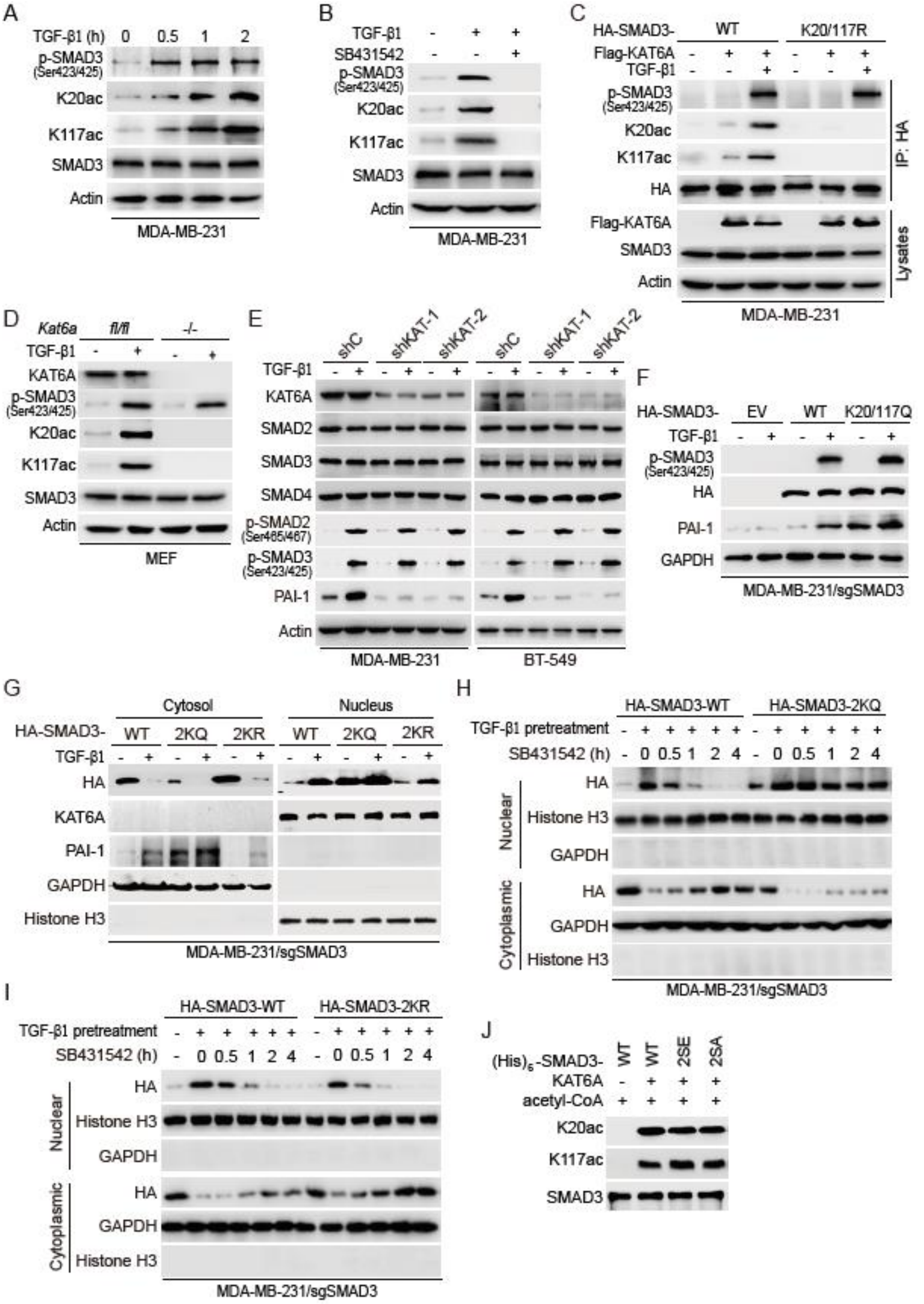

Supplementary Figure 3 KAT6A-acetylated SMAD3 depends on its phosphorylation. **A,** WB of SMAD3 phosphorylation (p-SMAD3), K20ac, and K117ac in MDA-MB-231 cells stimulated with TGF-β1 (5 ng/ml) as indicated time points. **B,** Effects of treatment of a TGF-β type I receptor inhibitor SB431542 on p-SMAD3, K20ac, and K117ac. MDA-MB-231 cells were pre-treated with or without SB431542 (10 μM, 4 h) and then

treated with or without TGF-β1 (5 ng/ml, 2 h). **C,** K20/117R mutation of SMAD3 did not impair TGF-β1-stimulated p-SMAD3. **D,** Effects of *Kat6a* knockout (KO) on p-SMAD3, K20ac, and K117ac. *Kat6a*$^{fl/fl}$ MEFs were transfected with Adeno-Cre virus to delete *Kat6a*. **E,** Effects of *KAT6A* KD on expression of SMAD2, SMAD3, SMAD4, p-SMAD2, p-SMAD3, and PAI-1 in MDA-MB-231 and BT-549 cells. **F**, Re-expression of SMAD3 K20/117Q (2KQ) mutant, but not WT SMAD3, rescued *SMAD3* KO-inhibited PAI-1 expression without TGF-β1 stimulation. **G**, WB of the subcellular localization of SMAD3 WT, 2KQ and 2KR mutants with or without TGF-β1 stimulation. MDA-MB-231/sgSMAD3 cells transfected with SMAD3 WT, 2KQ or 2KR mutant were stimulated with or without TGF-β1 (5 ng/ml) for 2 h. **H and I,** Compared to the WT, SMAD3 2KQ mutation delayed its nuclear export after SB431542 treatment whereas the 2KR mutation promoted its nuclear export. MDA-MB-231 cells were pre-treated with TGF-β1 (5 ng/ml) for 30 minutes. Then the cells were washed 3 times to remove TGF-β1 and treated with SB431542 (10 μM) for up to 4 hours. The cells were harvested at indicated time points, and both the nuclear and cytoplasmic fractions were collected. **J**, In vitro KAT analysis using recombinant active KAT6A and $(His)_6$-SMAD3 WT, 2SA (SSVS->SAVA, Ser423/425Ala) or 2SE (SSVS->SEVE, Ser423/425Glu) mutant protein. Acetylation of K20 or K117 was determined by using anti-K20ac or -K117ac antibodies, respectively. Data are representative of two independent experiments with similar results.

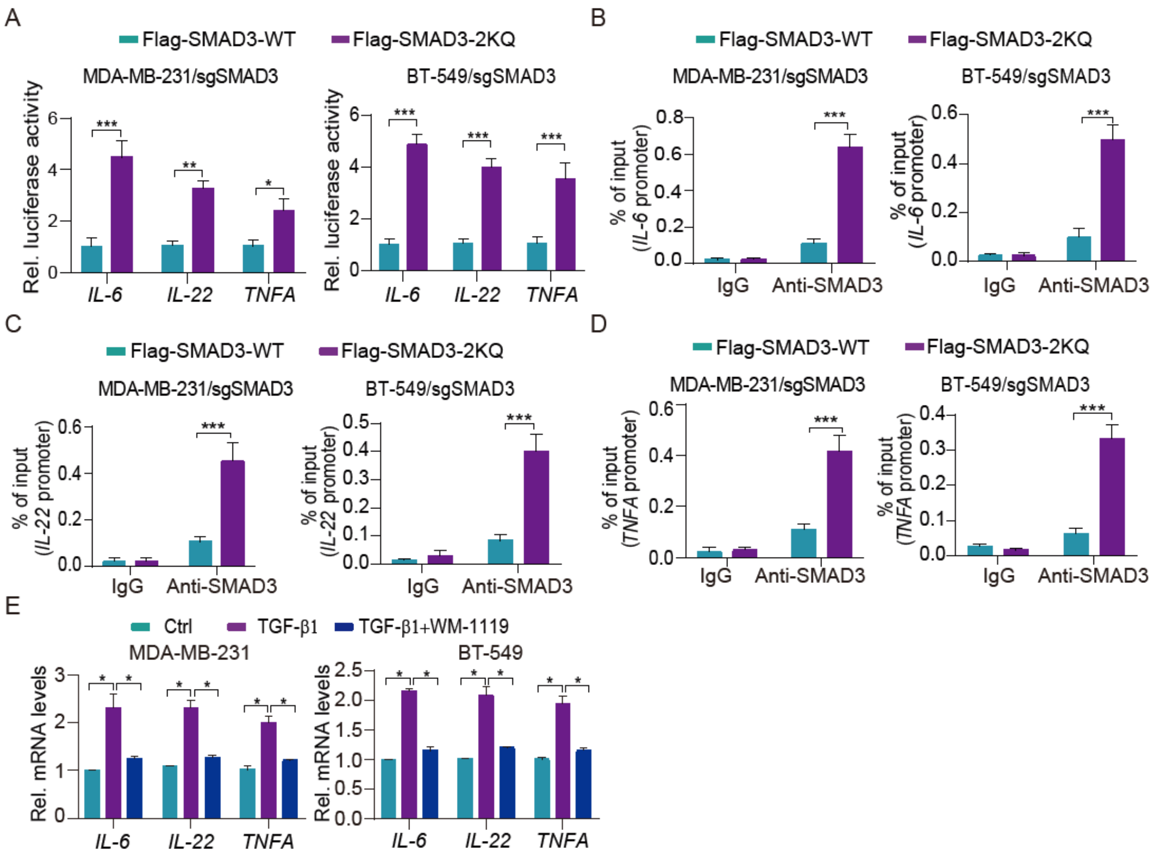

Supplementary Figure 4 K20/K117 acetylation of SMAD3 transcriptionally activates immune response-related cytokines. **A,** Effects of SMAD3-2KQ mutation on *IL-6, IL-22,* and *TNFA* promoter activity. A Dual-Luciferase reporter assay Kit (Promega) was used to measure Luciferase signals. **B-D,** ChIP-qPCR of effects of SMAD3-2KQ mutant on the binding of SMAD3 to *IL-6* (**B**), *IL-22* (**C**), and *TNFA* (**D**) promoters. **E,** qRT-PCR of TGF-β1-induced upregulation of *IL-6*, *IL-22*, and *TNFA* in MDA-MB-231 and BT-549 cells. Cells were pre-treated with or without KAT6A inhibitor WM-1119 and then treated with TGF-β1 (5 ng/ml, 1 h). Data are representative of three independent experiments with similar results. Error bars, SEM. *$P < 0.05$, **$P < 0.01$, and ***$P < 0.001$ by paired two-tail *t*-test.

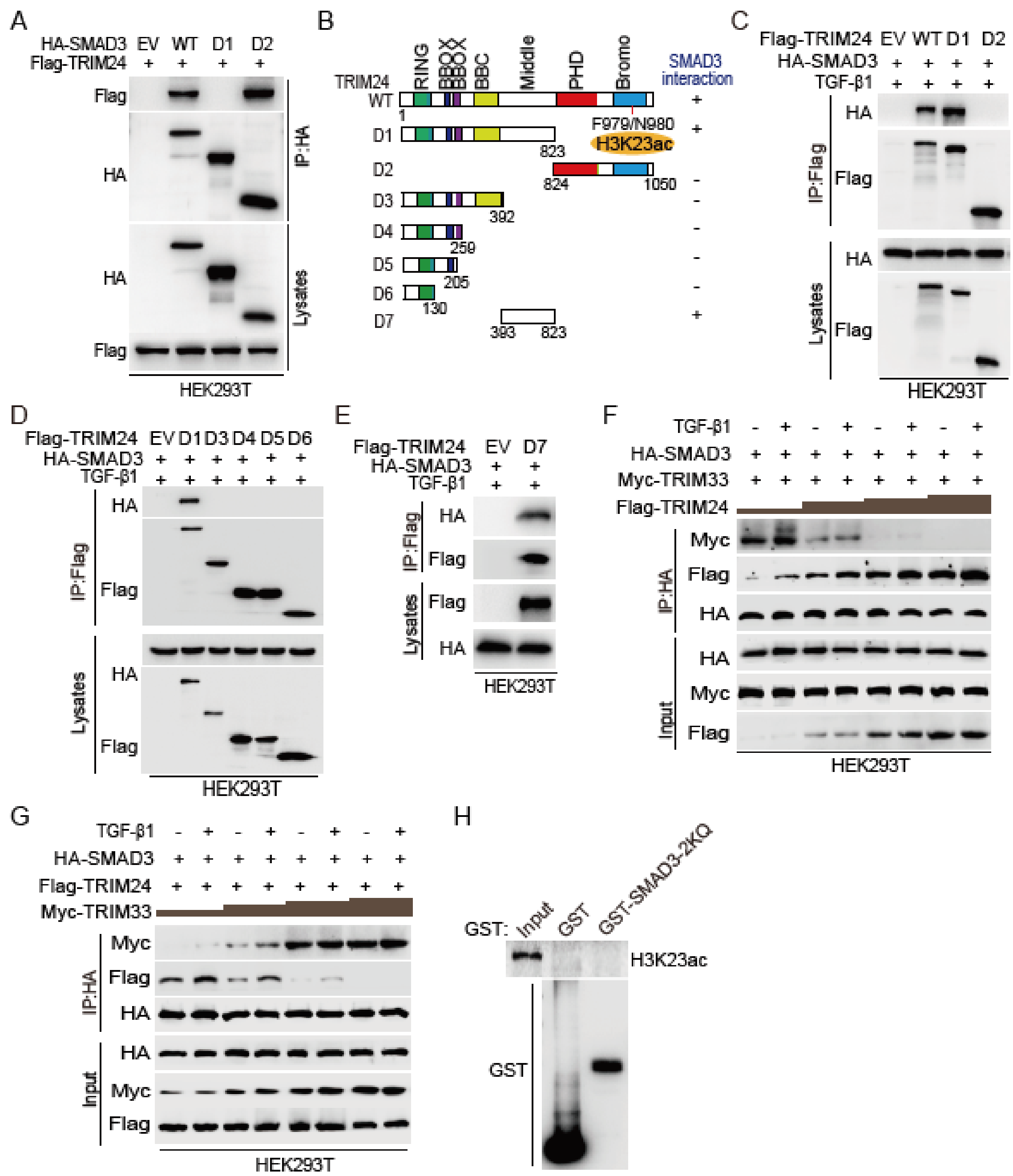

Supplementary Figure 5 TRIM24 competes with TRIM33 to interact with SMAD3. **A,** The MH2 domain (D2 mutant) of SMAD3 is required for SMAD3-TRIM24 interaction. **B,** Schematics of TRIM24 WT and various TRIM24 deletion mutants. **C** to **E,** IP and WB of TRIM24 truncated mutants with SMAD3 in HEK293T cells stimulated by TGF-β1. Flag-tagged TRIM24 WT and the truncated mutants were co-transducted into HEK293T cells with HA-SMAD3. EV, an empty vector control. **F,** TRIM24 overexpression enhanced its association with SMAD3 and disrupted TRIM33-SMAD3 interaction in HEK293T cells stimulated with TGF-β1. **G,** TRIM33 overexpression enhanced its association with SMAD3 and disrupted TRIM24-SMAD3 association in HEK293T cells stimulated with TGF-β1. Different amounts of Flag-tagged TRIM24 and Myc-TRIM33 were co-transfected into HEK293T cells with HA-SMAD3. **H,** In vitro GST pull-down analysis. Purified GST-SMAD3 2KQ mutant or GST control protein was incubated with recombinant

Histone H3K23ac (Expression Protein Ligation, EPL). Interaction between the 2KQ mutant and H3K23ac was analyzed with H3K23ac antibody. Data are representative of two independent experiments with similar results.

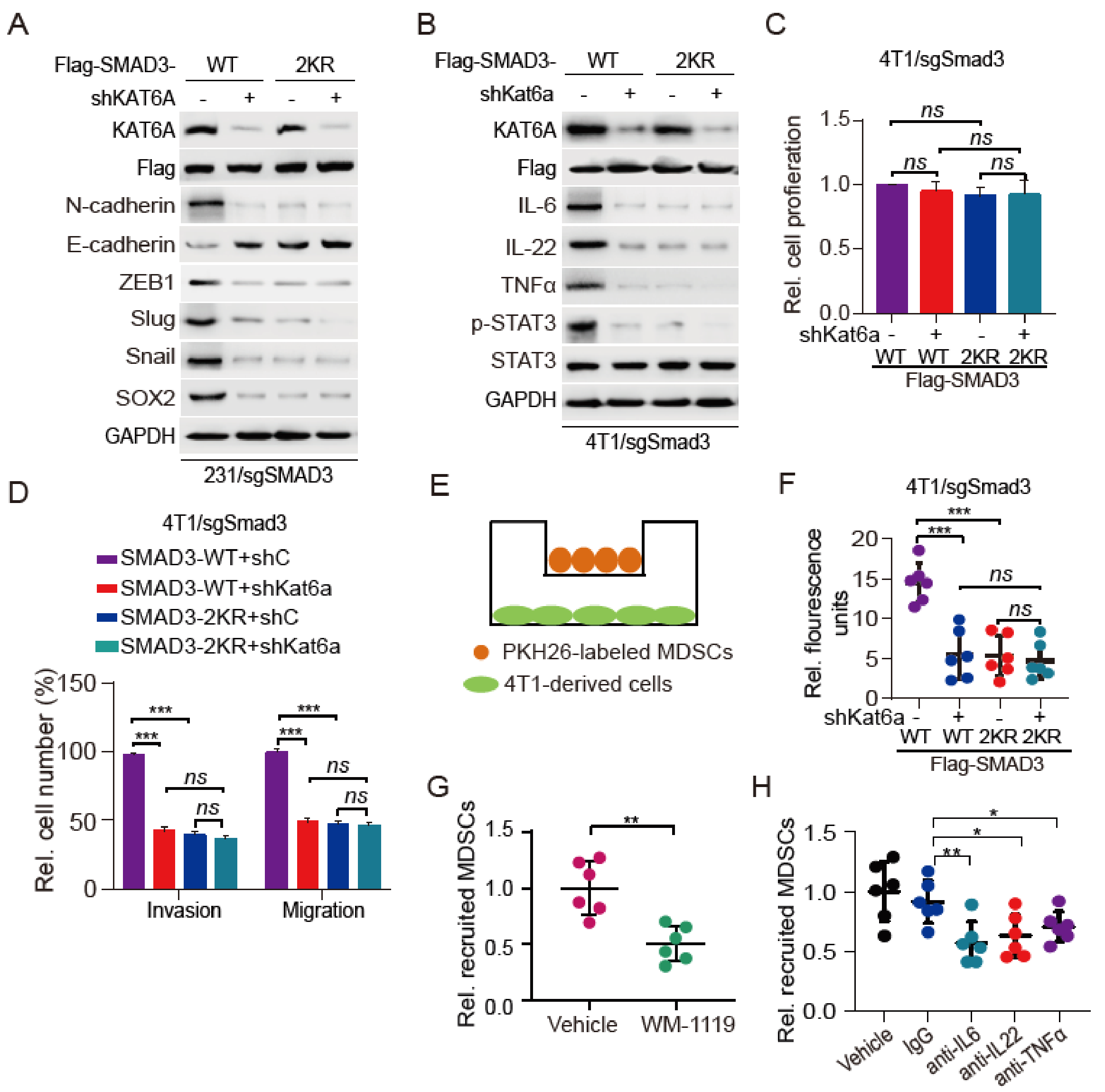

Supplementary Figure 6 Effects of SMAD3 2KR mutation on epithelial and mesenchymal transition (EMT)-related protein expression, cell proliferation, invasion, migration, and MDSC recruitment in vitro. **A,** Ectopic expression of SMAD3 2KR mutant decreased KAT6A-acetylated SMAD3-mediated EMT and CSCs-related protein expression in MDA-MB-231/sgSMAD3 cells. MDA-MB-231/sgSMAD3 stable cells were co- transfected with a KAT6A shRNA and Flag-SMAD3 WT or 2KR mutant. **B-D,** Compared with WT SMAD3, effects of further *Kat6a* KD or re-expression of SMAD3 2KR mutant on *Smad3* KO-inhibited expression of IL-6, IL-22, TNFα, and STAT3 phosphorylation (**B**), cell proliferation (**C**), invasion and migration (**D**). **E,** A schema of the migration experiment of MDSCs in vitro. **F,** Transwell migration assay of CD11b$^+$/Gr1$^+$ MDSCs attracted by various stable transfected 4T1 cells. **G** and **H**, Transwell migration analysis of MDSCs attracted by 4T1 breast cancer cells treated with a KAT6A inhibitor WM-1119 (**G**), an IgG, anti-IL6, anti-IL22, or anti-TNFα antibody (**H**). Rel., relative. Data is representative of three independent experiments with similar results. Error bars, SEM. *$P < 0.05$, **$P < 0.01$, ***$P < 0.001$ by paired two-tail t-test.

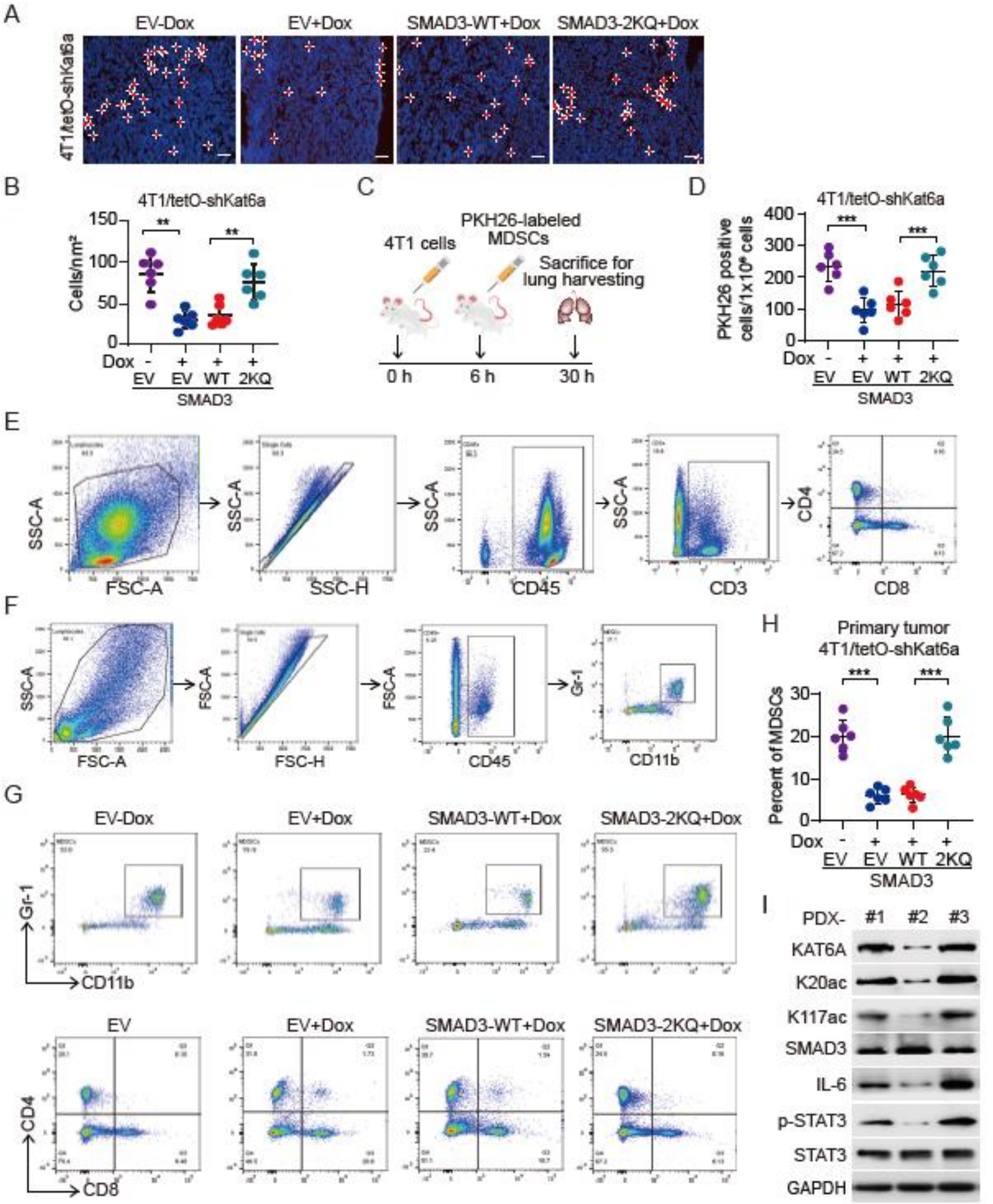

Supplementary Figure 7 The K20/117 acetylation of SMAD3 enhances MDSC recruitment in vitro and in vivo. **A,** Micrometastases analysis in lungs of BALB/c mice implanted with 1x 10^6 4T1-Luc2RFP cells via the tail vein injection. After 24 hours, mice were sacrificed and the lungs were harvested, fixed, stained with DAPI, and analyzed with a Nikon Laser Microscope System. **B,** Quantification of the 4T1-Luc2RFP cells in the lungs of the mice in **A** (n = 6). **C,** A schema for the animal experiment of recruiting MDSCs. **D,** Quantification of the recruited PKH26-labeled MDSCs in the lungs of the mice at 30 h after MDSC tail vein injections (n = 6). **E** and **F,** The FACS gating strategy for identification of $CD4^+$ T cells ($CD45^+CD3^+/CD4^+$), $CD8^+$ T cells ($CD45^+CD3^+/CD8^+$) (**E**) MDSCs ($CD45^+CD11b^+/Gr1^+$) (**F**) in the lungs of tumor-bearing mice. **G,** Representative FACS of MDSCs, $CD4^+$ T cells, and $CD8^+$ T cells in the lungs of indicated tumor-bearing mice. **H**, FACS of tumor-associated MDSCs ($CD45^+CD11b^+Gr1^+$) in the primary tumor from the orthotopic implantation assays in Figure 5F. **I,** WB of expression of KAT6A,

SMAD3-K20ac, -K117ac, IL6, and p-STAT3 in three TNBC breast PDX models. Data are representative of three independent experiments with similar results. Error bars, SEM. *$P < 0.05$, **$P < 0.01$, ***$P < 0.001$ by paired two-tail *t*-test.

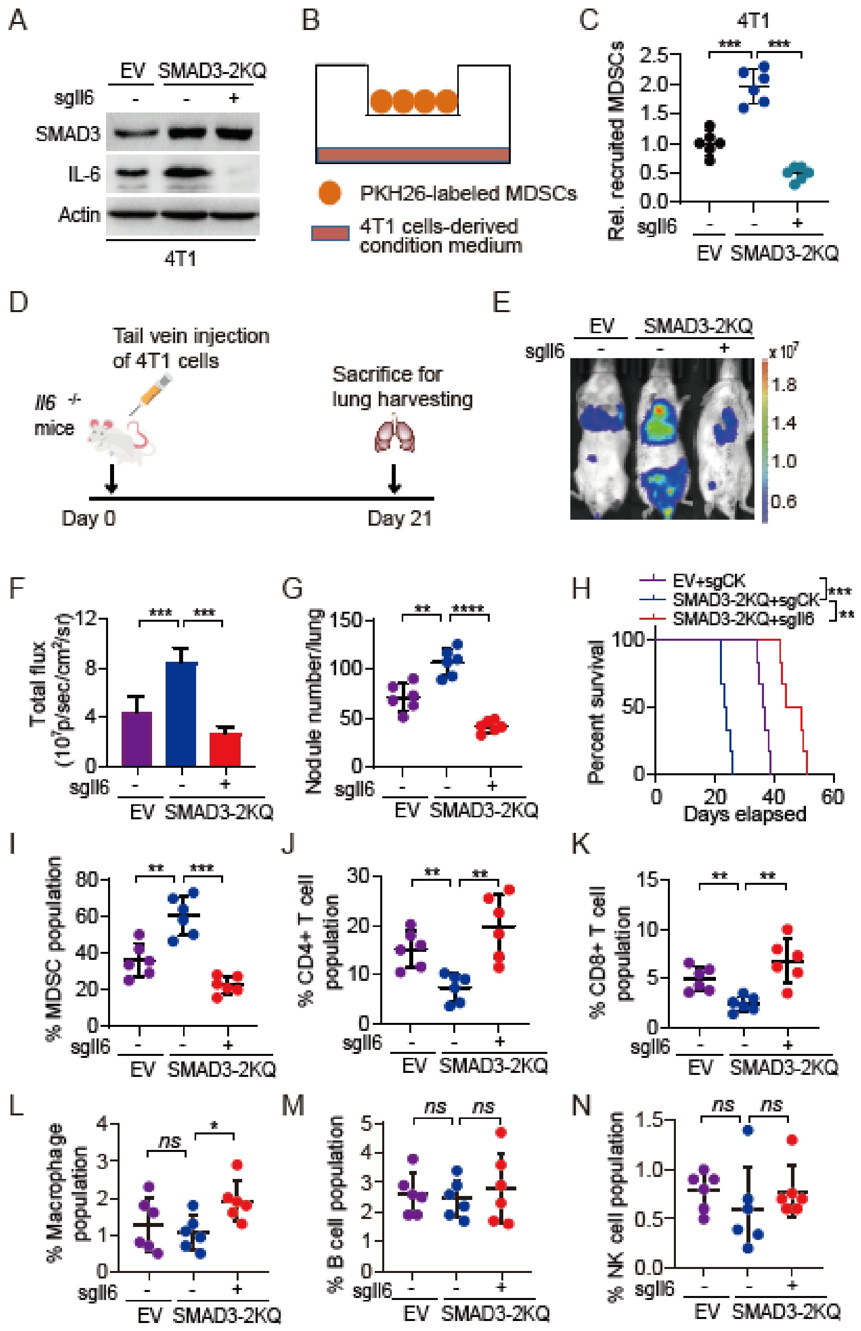

Supplementary Figure 8 Tumor-secreted IL6 is critical for SMAD3-induced tumor metastasis. **A,** WB of the effect of SMAD3 2KQ on IL6 expression in 4T1 breast cancer cells. EV, an empty vector control. sgIl6, a *Il6* sgRNA. **B,** Schema of the migration of MDSCs stimulated by *Il6* KO cell-derived condition medium. **C**, Quantification of MDSC migration stimulated by the condition medium derived from 4T1/sgIl6 cells. **D**, Schema of animal experiments. The 4T1 cells were injected into the *Il6*$^{-/-}$ mice through tail vein injection (n = 6). The mice were sacrificed for lung harvesting at 21 days after injection. **E,** Representative BLI images of tumor generation and metastases. **F,** Quantification of BLI in **E**. **G**, Quantification of number of surface lung metastases of mice in **E**. **H**, Kaplan-Meier survival analysis of animals bearing-4T1 tumors. **I-K**, FACS of the percentage of MDSCs (**I**), $CD4^+$ T cells (**J**), $CD8^+$ T cells (**K**) in the lungs of tumor-bearing mice. **L-N,** FACS of the percentage of (**L**) Macrophages ($CD45^+F4/80^+$), (**M**) B cells ($CD45^+CD19^+$), and (**N**) NK cells ($CD45^+CD49b^+$) in the lungs of indicated tumor-bearing mice. Data are representative of three independent experiments with similar results. Error bars, SEM. **P <0.01, ***P < 0.001 by paired two-tail t-test or log-rank analysis.

Supplementary Materials and Methods

KEY RESOURCES TABLE

REAGENT or RESOURCE	SOURCE	IDENTIFIER
**Antibodies**		
KAT6A	Abnova	H00007994-M07
TRIM24	Proteintech	14208-1-AP
TRIM33	Proteintech	55374-1-AP
GAPDH	Cell Signaling Technology	2118S
HA-Tag	Cell Signaling Technology	2367S
HA-Tag	Cell Signaling Technology	3724S
His-Tag	Cell Signaling Technology	12698S
Myc-Tag	Cell Signaling Technology	2276S
Acetylated-Lysine	Cell Signaling Technology	9441S
Acetyl-Histone H3 (Lys9)	Cell Signaling Technology	9649S
Acetyl-Histone H3 (Lys14)	Cell Signaling Technology	7627S
Acetyl-Histone H3 (Lys23)	Cell Signaling Technology	14932S
SMAD3	Cell Signaling Technology	9523S
Phospho-SMAD3 (Ser423/425)	Cell Signaling Technology	9520S
SMAD2	Cell Signaling Technology	5339S
Phospho-SMAD2 (Ser465/Ser467)	Cell Signaling Technology	18338S
SMAD4	Cell Signaling Technology	46535S
IL-6	Cell Signaling Technology	12153S
TNFα	Cell Signaling Technology	6945S
STAT3	Cell Signaling Technology	30835S

Phospho -STAT3 (Tyr705)	Cell Signaling Technology	9131S
IL-22	PEPROTECH	500-P211
ZEB1	Cell Signaling Technology	3396S
SOX2	Cell Signaling Technology	2748S
E-cadherin	Cell Signaling Technology	3195
N-cadherin	Cell Signaling Technology	13116
Slug	Cell Signaling Technology	9585
Snail	Cell Signaling Technology	3879
CD44	Cell Signaling Technology	37259S
Histone H3	Cell Signaling Technology	9715
β-Actin	Stanta Cruz	sc-47778
GST (B-14)	Stanta Cruz	sc-138
CD11b	Abcam	ab224805
CD4	Abcam	ab133616
CD8	Abcam	ab93278
PAI-1	Stanta Cruz	sc-5297
KAT6B	4A Biotech Co.,Ltd.	ABIN6388407
p300	Stanta Cruz	sc-32244
TIP60	Stanta Cruz	sc-81164
GCN5	Invitrogen	MA5-14884
PCAF	Stanta Cruz	sc-13124
lysine 20 acetylated SMAD3	Proteintech	/
lysine 117 acetylated SMAD3	Proteintech	/
**Biological Samples**		
Paraffin-embedded sections of breast cancer metastatic lymph nodes tissues	Ren Ji Hospital	Shanghai, China
**Chemicals, and Recombinant Proteins**		
SB431542	Selleck Chemicals	S1067
InVivoMAb anti-mouse PD-L1 (B7-H1)	Bio X cell	BE0101
WM1119	absin	abs821483
Doxycycline hyclate	Sigma	D9891
Recombinant KAT6A/MOZ(488-778) protein	Active Motif	81923

Recombinant Histone H3K23ac (EPL)	Active Motif	31255
Hieff TransTM Liposomal Transfection Reagent	YEASEN	40802ES08
**Critical Commercial Assays**		
GeneArt™ Site-Directed Mutagenesis PLUS System	Invitrogen™	A14604
Dual-Luciferase® Reporter Assay System	Promega	E1960
Human IL-6 Quantikine ELISA Kit	R&D systems	D6050
Human IL-22 Quantikine ELISA Kit	R&D systems	D2200
Human TNFα Quantikine ELISA Kit	R&D systems	DTA00D
SimpleChIP® Plus Enzymatic Chromatin IP Kit	Cell Signaling Technology	9005S
Cell Migration Assay (Fluorescence) kit	Biointersect	G8002-100
EasySep™ Mouse MDSC (CD11b+Gr1+) Isolation Kit	STEMCELLTM Technologies	Catalog #19867
ALDEFLUOR assay	STEMCELL Technologies	Catalog #01700
**Deposited Data**		
RNA-seq	This study	Deposited to GEO as GSE95386
**Experimental Models: Cell Lines**		
MDA-MB-231	The Chinese National Infrastructure of Cell Line Resource	Beijing, China
BT-549	The Chinese National Infrastructure of Cell Line Resource	Beijing, China
HEK-293T	The Chinese National Infrastructure of Cell Line Resource	Beijing, China
Kat6a$^{-/-}$ MEFs	This study	N/A
4T1	ATCC	CRL-2539
**Experimental Models: Organisms/Strains**		
Mouse: Pathogen-free female BALB/c	SLAC	Shanghai, China
Mouse: Athymic nude mice	SLAC	Shanghai, China
Mouse: CByJ.129S2(B6)-Il6^{tm1Kopf}/J	The Jackson Laboratory	Stock No: 007078
Mouse: BALB/cByJ	The Jackson Laboratory	Stock No: 001026
**Oligonucleotides**		

KAT6A-G657E-F:cagcgtaaggAAtatggcaggtttctcatcgatttc R:cctgccataTTccttacgctggtattgaggaagaat	Sangon Biotech	Shanghai, China
KAT6A-C543G-F:ttgtgaatttGgtctaaaatatatgaaaagtagaac R:tattttagacCaaattcacaaagatacaatttgggc	Sangon Biotech	Shanghai, China
SMAD3-K117R-F:aatatgaagaGggacgaggtctgcgtgaatccctacca R:cagacctcgtccCtcttcatattgaaggcgaactcaca	Sangon Biotech	Shanghai, China
SMAD3-K117Q-F:aatatgaagCaggacgaggtctgcgtgaatccctacca R:cagacctcgtcctGcttcatattgaaggcgaactcaca	Sangon Biotech	Shanghai, China
SMAD3-K20R-F:ggctggaagaGgggcgagcagaacgggcaggaggagaa R:ttctgctcgcccCtcttccagcccagcaggcgcttcac	Sangon Biotech	Shanghai, China
SMAD3-K20Q-F:ggctggaagCagggcgagcagaacgggcaggaggagaa R:ttctgctcgccctGcttccagcccagcaggcgcttcac	Sangon Biotech	Shanghai, China
SMAD3-S423E-F:ccgctgttccGAGgtgtctgggggtggaggctctta R:cccccagacacCTCggaacagcggatgcttggggagc	Sangon Biotech	Shanghai, China
SMAD3-S425E-F:ttccagtgtgGAGgggggtggaggctcttatcctta R:ctccacccccCTCcacactggaacagcggatgcttg	Sangon Biotech	Shanghai, China
SMAD3-S423A-F:ccgctgttccGCtgtgtctgggggtggaggctctt R:cccagacacaGCggaacagcggatgcttggggagc	Sangon Biotech	Shanghai, China

SMAD3-S425A-F:ttccagtgtgGctgggggtggaggctcttatcct R:ccacccccagCcacactggaacagcggatgcttg	Sangon Biotech	Shanghai, China
fhtUTG-KATA6-1-F:TCCCAGGAGGAGCCTGGTGTTCATTCAAGAGATGAACACCAGGCTCCTCCTTTTTTC	Sangon Biotech	Shanghai, China
fhtUTG-KATA6-1-R:TCGAGAAAAAAGGAGGAGCCTGGTGTTCATCTCTTGAATGAACACCAGGCTCCTCCT	Sangon Biotech	Shanghai, China
pGL3-IL-22-promoter-luciferase-F:TAGCCCGGGCTCGAGGTTAGACCTGCAGGCTAACACAGAC	Sangon Biotech	Shanghai, China
pGL3-IL-22-promoter-luciferase-R:CGGAATGCCAAGCTTGCCAAAGAAAGTCTTCACTCTGCTG	Sangon Biotech	Shanghai, China
pGL3-IL-6-promoter-luciferase-F:TAGCCCGGGCTCGAGACATCAGCTATGATGCAATCCAGCA	Sangon Biotech	Shanghai, China
pGL3-IL-6-promoter-luciferase-R:CGGAATGCCAAGCTTTCTTATTGGGTCAAGCTGGTTCAGA	Sangon Biotech	Shanghai, China
pGL3-TNFa-promoter-luciferase-F:TAGCCCGGGCTCGAGgcactcgatgtaccacgggg	Sangon Biotech	Shanghai, China
pGL3-TNFa-promoter-luciferase-R:CGGAATGCCAAGCTTcccggatcatgctttcagtgct	Sangon Biotech	Shanghai, China
TRIM24-FN979/980AA-F:gtgctgaaGCcGCtgagcctgattcagaagtagccaat	Sangon Biotech	Shanghai, China
TRIM24-FN979/980AA-R:tcaggctcaGCgGCttcagcacagttttgaaagatcaa	Sangon Biotech	Shanghai, China
pCDNA3.3-HA-TRIM33-F:AGAGAATTCGGATCCatggcggaaaacaaaggcggcgg	Sangon Biotech	Shanghai, China
pCDNA3.3-HA-TRIM33-R:CTTCCATGGCTCGAGTTACTTTATATGTACTGGTCTCTC	Sangon Biotech	Shanghai, China

pCDNA3.3-Flag-TRIM33-F:accggactcagatctcgagGCCACCATGGACTACAAAG	Sangon Biotech	Shanghai, China
pCDNA3.3-Flag-TRIM33-R:agagtcgcggatccTTTAAGCAACTGGCGttctt	Sangon Biotech	Shanghai, China
KAT6A-SH1-F:tGCCCATCGTTTCCATTCCTAACTCGAGTTAGGAATGGAAACGATGGGCTTTTTc	Sangon Biotech	Shanghai, China
KAT6A-SH1-R:tcgagaaaaaGCCCATCGTTTCCATTCCTAACTCGAGTTAGGAATGGAAACGATGGGCa	Sangon Biotech	Shanghai, China
KAT6A-SH3-F:tGCTCTGAAGTGCAGATTAACACTCGAGTGTTAATCTGCACTTCAGAGCttttc	Sangon Biotech	Shanghai, China
KAT6A-SH3-R:tcgagAAAAGCTCTGAAGTGCAGATTAACACTCGAGTGTTAATCTGCACTTCAGAGCa	Sangon Biotech	Shanghai, China
KAT6A-SH5-F:TGCAACATGTCTGCCACCAACACTCGAGTGTTGGTGGCAGACATGTTGCttttc	Sangon Biotech	Shanghai, China
KAT6A-SH5-R:tcgagAAAAGCAACATGTCTGCCACCAACACTCGAGTGTTGGTGGCAGACATGTTGCa	Sangon Biotech	Shanghai, China
qRT-PCR,GAPDH:5’-GGAGCGAGATCCCTCCAAAAT-3’ and 5’-GGCTGTTGTCATACTTCTCATGG-3’	Sangon Biotech	Shanghai, China
qRT-PCR, IL-6:5’-TTCTCCACAAGCGCCTTC-3’ and 5’-AGAGGTGAGTGGCTGTCTGT-3’	Sangon Biotech	Shanghai, China
qRT-PCR, IL-22:5’-GACAAGTCCAACTTCCAG -3’ and 5’-GCTCACTCATACTGACTC -3’	Sangon Biotech	Shanghai, China
qRT-PCR, TNFα:5’-TGCACTTTGGAGTGATCGGC-3’ and 5’-CTCAGCTTGAGGGTTTGCTAC-3’	Sangon Biotech	Shanghai, China
**Recombinant DNA**		

PCDNA3.3-KAT6A WT and mutants	This study	N/A
pLVX-Puro-KAT6A	This study	N/A
pCMV3-SMAD2	This study	N/A
pCMV3-SMAD3 WT and mutants	This study	N/A
pCMV3-SMAD4	This study	N/A
pLVX-Puro-SMAD3 WT and mutants	This study	N/A
PCDNA3.3-TRIM24 WT and mutants	This study	N/A
PCDNA3.3-TRIM33	This study	N/A
psPAX2	Addgene	82416
pCMV-VSV-G	Addgene	12260
pGL3.0-basic-IL-6 promoter	This study	N/A
pGL3.0-basic-IL-22 promoter	This study	N/A
pGL3.0-basic-TNFA promoter	This study	N/A
pGEX-4T-1-KAT6A	This study	N/A
GIPZ shRNA Control	Dharmacon	Cat#RHS4346
KAT6A shRNA#1	Shanghai Jiao Tong University	V3LHS_397899
KAT6A shRNA#3	Shanghai Jiao Tong University	V2LHS_190181
KAT6A shRNA#5	Shanghai Jiao Tong University	V2LHS_84566
**Software and Algorithms**		
shRNA design MIT on line tool		http://crispr.mit.edu
cbioportal		http://www.cbioportal.org
**Antibody and auxiliaries used for flow cytometry Antibody**		
CD45	557659	BD Pharmingen™ APC-Cy™7 Rat Anti-Mouse CD45
CD3	553061	BD Pharmingen™ FITC Hamster Anti-Mouse CD3e
CD4	563151	BD Horizon™ BV605 Rat Anti-Mouse CD4
CD8	552877	BD Pharmingen™ PE-Cy™7 Rat Anti-Mouse CD8a
CD11B	562605	BD Horizon™ BV421 Rat Anti-CD11b
GR1	553129	BD Pharmingen™ APC Rat Anti-Mouse Ly-6G and Ly-6C
CD11c	550261	BD Pharmingen™ APC

		Hamster Anti-Mouse CD11c
CD19	561740	BD Pharmingen™ FITC Rat Anti-Mouse CD19
CD49b	553858	BD Pharmingen™ PE Rat Anti-Mouse CD49b
F4/80	565410	BD Pharmingen™ PE Rat Anti-Mouse F4/80
FC BLOCK	553141	BD Pharmingen™ Purified Rat Anti-Mouse CD16/CD32 (Mouse BD Fc Block™)
Red Blood Cell Lysis Buffer	555899	BD Pharm Lyse™ Lysing Buffer

EXPERIMENTAL MODEL AND SUBJECT DETAILS

Animal Xenograft Studies

For the orthotopic xenograft model, 4T1 cells (5×10^5) were suspended in 50 µl of PBS and mixed with matrigel (1:1), and then were injected into the fourth mammary fat pad of BALB/c mice following our established protocol [1] (6 mice for each group). For the tumor metastasis model, MDA-MB-231 (1×10^6), or 4T1 (5×10^5) in 100 µl of PBS were injected through tail vains or the fourth mammary fat pad. Bioluminescence (BLI) imaging was performed using the IVIS Lumina imaging station (Caliper Life Sciences). The mice were then sacrificed, and the tumor, lung, or spleen tissues were harvested for measuring MDSC, $CD4^+$ and $CD8^+$ T-cells, Macrophages, B-cells, and NK-cells infiltration. MDSCs were stained with MDSC-specific surface markers and isolated with a FACSAria III instrument (BD Biosciences) after cell isolation using a Ficoll density gradient (GE Healthcare) to remove granulocytes. In drug treatment experiments, mice were treated

with WM-1119 (abs821483, Absin) and/or an anti-PD-L1(B7-H1) antibody (BE0101, BioXCell) at the set concentration on the alternating days.

Plasmid construction

KAT6A, SMAD2, SMAD3, SMAD4, and TRIM33 cDNAs were amplified by PCR and then cloned into a pcDNA3.3 or pLVX-Puro vector (Clontech), respectively. TRIM24 was constructed as previous described.[2] Point mutations were generated using a site-directed mutagenesis kit (Invitrogen) following the manufacturer's protocol. TRIM24 or SMAD3 truncated constructs were generated from pcDNA3.3-TRIM24 or pCMV3-SMAD3. GST-KAT6A and GST-SMAD3 were inserted into a pGEX-4T-1 vector for expression in *E*. coli. *IL-6*, *IL-22*, and *TNFA* promoter, extending from -2000 to +200 relative to the transcription start site, was cloned into a pGL3.0-basic luciferase reporter vector.

shRNA-knockdown, sgRNA-knockout, and transfection assays

KAT6A shRNAs were purchased from Genechem, Inc (Shanghai, China). Single-guide RNA (sgRNA) sequences of KAT6A were designed using the online tool from the MIT online tool (http://crispr.mit.edu). Targeted DNAs and packaging plasmids were transfected into the HEK293T cells using the Hieff Trans^{TM} Liposomal Transfection Reagent (40802ES08, YEASEN) following the manufacture’s instruction. The supernatants were collected and filtered at 48 and 72 hours after transfection. Viruses were then concentrated. Targeted Cells were infected with 8 μg/ml polybrene

(Sigma-Aldrich). Infected cells were selected with puromycin after infection. Multiple monoclonal cultures were screened for sgRNA by Western blotting and RT-PCR analyses.

immunoprecipitation (IP) and Western blotting (WB) assays

IP and WB assays were performed as previously described.[2] In brief, cells were lysed in IP lysis buffer (20 mM Tris-HCl pH 7.5, 1% Triton X-100, 150 mM NaCl, 2 mM Na_3VO_4, 5 mM NaF, and 1 mM EDTA) supplemented with complete protease inhibitor cocktail (Roche) at 4°C for 30 min. The lysates were cleared by centrifugation and immunoprecipitated with specific antibodies and protein G- G-agarose beads (Invitrogen). Proteins were separated by SDS-PAGE gels and visualized by enhanced chemiluminescence (ECL, Bio-Rad) reaction according to the manufacturer’s instructions. Cell fractionation was conducted using Subcellular Protein Fractionation Kit (Pierce).

Purification of recombinant proteins and GST pull-down assay

Recombinant GST-conjugated KAT6A or SMAD3 was generated by transforming the *E. coli* BL21 with pGEX-4T-1-KAT6A or pGEX-4T-1-SMAD3 and purified using glutathione Sepharose 4B beads (GE Healthcare) according to the manufacturer’s protocol. $(His)_6$-TRIM24 or $(His)_6$-SMAD3 plasmid was transduced into HEK293T cells, and cells were lysed. $(His)_6$-TRIM24 or $(His)_6$-SMAD3 protein in the supernatant were then purified using a Ni^+-NTA column. Recombinant Histone H3 acetyl Lys23 (H3K23ac) proteins generated using expressed protein ligation (EPL) technology were purchased from the

Active Motif (Catalog No:31255).

Proteomics Analysis

Proteomics analyses for KAT6A-associated proteins and SMAD3 acetylation were performed at Jiyun Biotech.Inc (Shanghai, China). Briefly, MDA-MB-231 cells expressing Flag-tagged KAT6A or an empty vector control were lysed, and the supernatants were immunoprecipitated with the anti-FLAG M2 Magnetic Beads (MCE, Cat. No. M8823). Then, the immunoprecipitants were washed three times with wash buffer (50 mM Tris pH 7.5, 150 mM NaCl, 1 mM EDTA, 0.5% NP40, 10% glycerin) and bead-bound proteins were eluted with wash buffer plus 500 µg /ml FLAG peptides. The samples were sent to Jiyun Biotech.Inc and analyzed as previous described.[3] Unique peptides that were detected only in Flag-KAT6A immunoprecipitants, or displayed at least 2-fold higher abundance than the empty vector control groups, were selected. From all these peptides, only the ones that emerged in all of the three replicates were considered as KAT6A associated proteins.

RNA-Seq analysis and gene set enrichment analysis

RNA-Seq and differentially expressed gene analysis were performed as previously described[2]. Gene set enrichment analysis (GSEA) was conducted using GSEA2.2.4 software to generate enrichment scores for gene sets in Hallmark C2.all, C5.all, and C6.all data sets with default settings. Gene sets were tested for enrichment in rank-ordered lists via GSEA using a weighted Kolmogorov-Smirnov–like statistic to

calculate the enrichment score. The RNA-Seq data were deposited in the GEO database with accession number GSE95386.

Luciferase promoter activity assay

pGL3.0 basic-*IL-6*, *IL-22* and *TNFA* promoter was performed co-transfection with or without SMAD3 WT or K20/117R mutant using Lipofectamine™ 3000 Transfection Reagent (Thermo Fisher) according to the manufacturer's protocol. A pRL Renilla luciferase control reporter vector (Promega) was utilized as a negative control. A dual-luciferase assay was analyzed 48 h after co-transfection using the Promega E1960 Dual-Luciferase® Reporter System following the manufacturer's recommendation.

Chromatin immunoprecipitation (ChIP) and quantitative PCR (qPCR)

ChIP analysis was performed using a SimpleChIP® Plus Enzymatic Chromatin IP Kit (Magnetic Beads, Cell Signaling Technology, 9005S). Cells were harvested and cross-linked with 1% formaldehyde. Cell nuclei were prepared and chromatin was incubated with Micrococcal Nuclease 37°C for 20 min, followed by appropriate sonication. The supernatants were immune-precipitated using 3 μg anti-SMAD3 antibody or the relevant non-specific IgG at 4°C for 16 h. ChIP DNA was purified and subsequently quantified by qPCR.

Cell invasion and migration analysis

MDA-MB-231 or BT-549 cells (5×10^4) suspended in medium without FBS were plated on the upper chamber membranes (8 µm pore size, Corning) coated with Matrigel (BD Biosciences). The inserts were incubated in medium supplemented with 10% FBS for 16 h. To evaluate the invasion ability, non-invasive cells were removed. Invasive cells were fixed with methanol, stained with crystal violet, and counted. The MDSCs migration was detected by a Cell Migration Assay (Fluorescence) kit (biointersect, Catalog # G8002-100) following the manufacturer's recommendation.

Flow cytometry analysis (FACS) and sorting

Single-cell suspensions were prepared and incubated on the ice with a combination of antibodies for 30 min in the dark. FACS analysis was performed using the LSRII Flow Cytometer (BD Biosciences), and data were analyzed using the FlowJo software (Tree Star Inc.). For FACS sorting, Aria II or FACS Jazz instruments were used.

$ALDH^+$ cell staining

The $ALDH^+$ cell staining was performed by using a ALDEFLUOR assay kit according to the manufacturer's guidelines (STEMCELL Technologies). Briefly, cells were suspended in ALDEFLUOR assay buffer containing an ALDH substrate, BODIPY-aminoacetaldehyde (BAAA), and incubated for 30 min at 37°C. Cells treated with diethylaminobenzaldehyde, an ALDH inhibitor, were used as control. The result in fluorescence intensity of $ALDH^+$ cells was analyzed by flow cytometer (Beckman Coulter, CytoFLEX). Propidium iodide (PI) staining was used to identify and gate out dead and

late apoptotic cells.

Mammosphere formation and extreme limiting dilution assay (ELDA)

The single-cell suspension was obtained by trypsinization and sieving through a 40-mm sieve. Single cells were plated in ultralow attachment six-well plates (Corning, 3471) at a density 1×10^3 cells/well and cultured in sphere medium (DMEM/F12, 20 ng/ml epidermal growth factor (EGF), 20 ng/ml basic fibroblast growth factor (bFGF), 20 µl/ml B27, and 4 mg/ml heparin for 10 days. Then, mammospheres were photographed under an inverted microscope (10× objective, Olympus).

For ELDA, cells were seeded into 96-well ultralow attachment plates with sphere medium at density of 5, 10, 20, 50, 100 cells/well (12 wells per cell density). After 10 days, each well was examined for the formation of tumor spheres. Stem cell frequency was calculated using extreme limiting dilution analysis (http://bioinf.wehi.edu.au/software/elda/).

Immunohistochemical staining (IHC)

The tissue sections from paraffin-embedded de-identified human breast cancer metastatic lymph node specimens were stained with anti-KAT6A (1:50), anti-SMAD3-K20ac (1:10), anti-K117ac (1:10), anti-CD11b (1:100), anti-CD4 (1:500), anti-CD8 (1:350) antibodies. Non-specific IgG was used as negative control. IHC staining was scored as 0-7 according to the percentage of positive cells as previously described

(sang 2018). Tumors with 0 or 2 staining scores were considered as low expressing and those with scores of 3-7 were considered high expressing. The stained tissues were scored by two individuals blinded to the clinical parameters.

References

[1] J. Tang, Y. Li, Y. Sang, B. Yu, D. Lv, W. Zhang, and H. Feng, *Oncogene*. **2018**, 37, 4723.

[2] D. Lv, Y. Li, W. Zhang, A. A. Alvarez, L. Song, J. Tang, W. Q. Gao, B. Hu, S. Y. Cheng, and H. Feng, *Nat Commun*. **2017**, 8, 1454.

[3] R. Chen, Y. Liu, H. Zhuang, B. Yang, K. Hei, M. Xiao, C. Hou, H. Gao, X. Zhang, C. Jia, L. Li, Y. Li, and N. Zhang, *Nucleic Acids Res*. **2017**, 45, 9947.
